# Defect-rich CeO_2−*x*_ nanosheets as efficient oxidase nanozymes for colorimetric Hg^2+^ detection and visible-light photocatalysis

**DOI:** 10.1039/d6ra02432g

**Published:** 2026-07-02

**Authors:** Ashish Kumar, Suchi Smita Singh, Amit Pathak, Suverna Trivedi, Debanjan Guin, Chandra Shekhar Pati Tripathi

**Affiliations:** a Department of Physics, Institute of Science, Banaras Hindu University Varanasi 221005 Uttar Pradesh India tripathi.csp@bhu.ac.in; b Department of Chemistry, Institute of Science, Banaras Hindu University Varanasi 221005 Uttar Pradesh India debanjan.chem@bhu.ac.in; c Department of Chemical Engineering, Indian Institute of Technology Kharagpur Kharagpur 721302 India strivedi@che.iitkgp.ac.in

## Abstract

Defective, oxygen vacancy-rich CeO_2−*x*_ nanosheets were synthesised using a simple two-step process. The synthesised CeO_2−*x*_ nanosheets exhibit excellent oxidase–mimic activity compared with their CeO_2_ nanosheet counterparts. Color change from colorless to red in aqueous dopamine solution and colorless to green coloration in aqueous 2,2′-azino-bis(3-ethylbenzothiazoline-6-sulphonic acid)diammonium salt (AzBTS) solution by CeO_2−*x*_ nanosheets confirms the creation of oxygen vacancies in the CeO_2_ nanosheets. Again, Raman and XPS analysis of CeO_2−*x*_ nanosheets confirms a higher concentration of Ce^3+^ ions and lower oxidant content than CeO_2_ nanosheets. The band gaps of CeO_2−*x*_ and CeO_2_ nanosheets were found to be 2.82 eV and 2.97 eV, respectively. The remarkable oxidase mimic activity can strongly catalyze the oxidation of chromogenic substrates like 3,3′,5,5′-tetramethylbenzidine (TMB) into a blue-colored cation radical without the need for an additional oxidizing agent. As mercury(ii) (Hg^2+^) ions are recognised as one of the most toxic metal ions in the world and exposure to Hg^2+^ ions, even in low concentration, can severely affect living organisms, here we detect Hg^2+^ ions in drinking water by a simple colorimetric detection method. Blue colored solution of oxidized TMB (ox-TMB) in the presence of glutathione can be converted to colorless TMB. Whereas, a blue-coloured solution of ox-TMB in the presence of both glutathione and Hg^2+^ ions can effectively be recovered. Based on this phenomenon, a simple and rapid colorimetric method for sensing Hg^2+^ ions with the naked eye was developed, showing a good linear response from 1 to 17.4 µM. The detection limit was estimated at 24.92 nM. Key operational parameters, including buffer composition, selectivity, pH, reaction time, and scavenger study, were established. The proposed method was successfully applied for Hg^2+^ detection in real water samples with good recovery results. In addition to the oxidase mimic Hg^2+^ ion sensing activity, a comparative photocatalytic efficiency of CeO_2_ nanosheets and CeO_2−*x*_ nanosheets was also assessed by evaluating the degradation of methylene blue dye under visible light irradiation.

## Abstract 2

Defective, oxygen vacancy-rich CeO_2−*x*_ nanosheets were prepared. CeO_2−*x*_ nanosheets exhibit superior oxidase-mimic activity, enabling oxidant-free oxidation of chromogenic substrates. The material was successfully applied for highly sensitive naked-eye Hg^2+^ detection and dye degradation.

## Graphical abstract

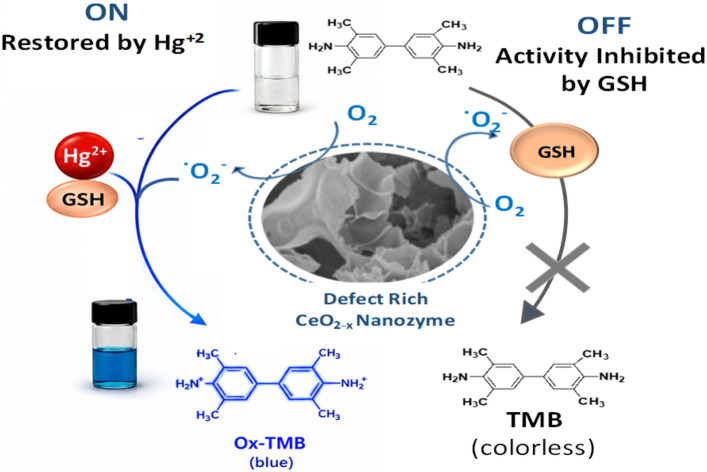

## 1. Introduction

Biocompatible cerium oxide (CeO_2_) has attracted significant attention in different fields such as catalysis, solar cells, oxygen sensors, and biological applications due to its unique redox properties. Along with these applications, CeO_2_ displays multiple enzyme mimetic activities with good free radical scavenging activity.^1–4^ It is reported that the increase in oxygen vacancies in CeO_2_ enhances the oxygen transport/storage ability, resulting in an improvement in its enzyme-mimic activities.^5^ Consequently, various strategies have been employed to enhance the oxygen vacancies, especially on the surface of CeO_2_, including altering morphology, reducing particle size, doping with metals, combining with other materials, and reducing with hydrogen.^6–8^

Modern globalisation and industrial development have led to highly polluted living conditions for humans, with water sources, essential to life on Earth, being the most severely affected. Heavy metals are among the most potent pollutants compared to many other contamination sources. They can enter the human body through contaminated food, drinking water, or air, posing serious health risks due to their high toxicity and ability to bioaccumulate.^9,10^ Mercury (Hg) is considered as one of the most toxic heavy metal.^11^ Exposure can lead to severe health problems, including kidney failure, motor dysfunction, and brain damage.^12,13^ Therefore, detecting Hg^2+^ ions in the environment, especially in drinking water at concentrations that affect human health and with good sensitivity, remains of vital importance.

To date, several analytical methods and techniques have been developed to monitor Hg^2+^ ion level in the environment, including atomic absorption spectroscopy,^14^ high-performance liquid chromatography,^15^ chemiluminescence, fluorometric^16,17^ and colorimetric methods. Most of these are expensive, time-consuming, and require sophisticated instrumentation and rigorous sample preparation techniques, which limit their practical and field applications. Meanwhile, it is crucial to monitor Hg^2+^ levels in the environment, especially in water bodies. The colorimetric sensing approach, among the above-mentioned methods, has been considered highly appealing due to its rapid response, high sensitivity, affordability and advantage of on-field application. In the past, highly sensitive, selective and label-free noble metal-based functionalized colorimetric techniques for the detection and speciation analysis of trace heavy metals were developed. Nanomaterial-based colorimetric platforms have emerged as powerful analytical tools for sensing applications, enabling visual detection of sensing events through distinct color changes.^18–22^

Nanozymes, nanomaterials with enzyme-like activity, exhibiting diverse enzyme-mimetic behaviour have been assessed for a range of sustainable applications, including biosensing, diagnostics, synergistic cancer therapy, and the detection of disease biomarkers and pollutants.^23,24^ First reported in 2009, CeO_2_ nanoparticles, possessing intrinsic oxidase–mimic activity, can oxidize small molecules, organic dyes, and chromogenic substrates without an external oxidizing agent.^25^ In recent years, nanostructured CeO_2_ with tunable oxygen vacancies has attracted considerable interest as an efficient redox nanocatalyst and has been widely explored for catalytic applications. The Ce^3+^/Ce^4+^ redox cycle, closely associated with surface oxygen vacancies, enhances charge transport and oxygen activation, thereby playing a critical role in catalytic activity during oxidation processes.^26–30^ Oxygen vacancies can activate the physically adsorbed molecular oxygen over the catalyst surface and transform it into reactive oxygen species (ROS), playing a critical role in oxidation reactions.^31^

Literature survey, Table S3, shows that many reported colorimetric detection systems rely on the use of noble metals, complex hybrid nanostructures or H_2_O_2_-assisted peroxidase mechanisms for Hg^2+^. The H_2_O_2_-free catalytic mechanism can minimise interference associated with peroxide instability, a noble metal-free single-component catalyst can reduce the overall cost and avoid complex synthesis steps, thereby improving operational simplicity and enhancing suitability for practical sensing applications. Herein, we have developed a simple, cost-effective and scalable synthesis method to produce oxygen-vacancy-rich 2D CeO_2_ nanostructures for the first time. Oxygen vacancies in sugar-blown-synthesised CeO_2_ nanosheets (CeO_2_ NSs) were introduced by heating with NaBH_4_ in an N_2_ atmosphere. A series of physicochemical characterizations confirmed the creation of oxygen vacancies. The oxygen-vacancy-rich CeO_2−*x*_ nanosheets (CeO_2−*x*_ NSs) showed superior oxidase–mimetic activity compared to pristine CeO_2_ NSs. CeO_2−*x*_ NSs catalyze the oxidation of a series of chromogenic substrates, namely dopamine (DA), 2,2′-azino-bis(3-ethylbenzothiazoline-6-sulphonic acid)diammonium salt (AzBTS), and 3,3′,5,5,5′-tetramethylbenzidine (TMB), with enhanced catalytic activity compared to their CeO_2_ NSs counterpart. Therefore, utilising the superior oxidase mimic activity of CeO_2−*x*_ NSs, we have developed a novel off–on colorimetric sensor for Hg^2+^ ions in aqueous media. A series of reaction parameters was optimised to obtain the best sensor response. Under optimal reaction conditions, the developed colorimetric sensor demonstrated a broad linear range (1–17.4 µM) of applicability with a limit of detection of 24.92 nM. The sensor was tested on real water samples and showed selective sensing of Hg^2+^ ions, with good recovery. Along with this visible-light-driven photocatalytic degradation of methylene blue dye using CeO_2−*x*_ NSs, and CeO_2_ NSs was also performed and compared. The degradation results show that the CeO_2−*x*_ NSs can perform catalytic activity even under visible-light irradiation with enhanced efficiency. Therefore, the multifunctional nature of CeO_2−*x*_ NSs, combining oxidase-like sensing and photocatalytic activity, highlights their potential for environmental monitoring and remediation applications.

## 2. Experimental section

### 2.1. Synthesis of CeO_2−*x*_ NSs

**Scheme 1:**
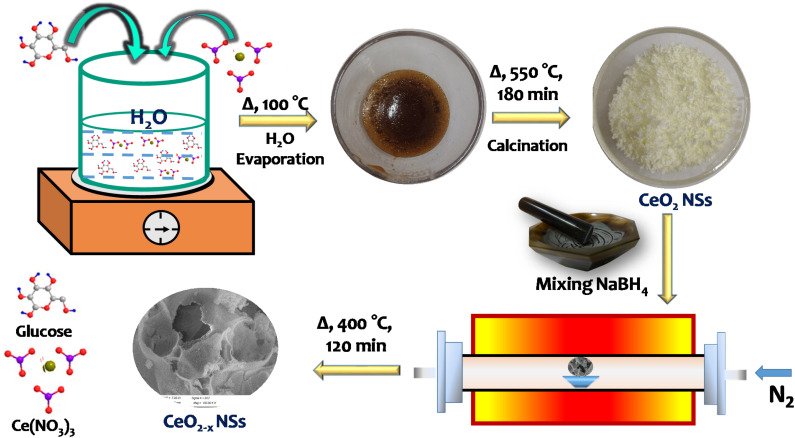
Synthesis of CeO_2_ NSs and CeO_2−*x*_ NSs.

CeO_2−*x*_ NSs were synthesized in two steps. Initially, CeO_2_ NSs were prepared using a simple, one-step, cost-effective sugar-blowing method.^32^ In the standard procedure, 5 mmol of Ce(NO_3_)_3_·6H_2_O and 10 mmol of glucose were added to DW and stirred magnetically to produce a clear, transparent solution. This solution was then heated on a hot plate to evaporate the DW until a brown colored molten syrup formed. The syrup was calcined at 550 °C with a heating rate of 10 °C min^−1^ for 3 hours in air. The resulting aerogel-like structure was ground with a mortar and pestle to get a yellow-colored CeO_2_ NSs powder sample. Then, CeO_2−*x*_ NSs were obtained by chemically reducing CeO_2_ NSs with NaBH_4_. In brief, CeO_2_ NSs and NaBH_4_ were thoroughly mixed in the mass ratio of 1 : 4 for this reduction reaction using a mortar and pestle. The mixture was transferred to an alumina boat and heated at 400 °C for 2 h in a two-zone tubular furnace, with a heating rate of 2 °C min^−1^ under controlled N_2_ flow. The resulting gray-colored CeO_2−*x*_ NSs were purified by three successive washings with DW and ethanol through centrifugation and decantation, followed by drying at ambient temperature. The step-by-step synthesis procedure is depicted in Scheme 1.

### 2.2. Evaluation of oxidase mimic activity

The oxidase mimic activities of CeO_2_ NSs and CeO_2−*x*_ NSs were measured and compared, as detailed in our previous work.^32^ In brief, a known amount of catalyst was dispersed in 2 mL citric acid buffer solution and taken in three different sample vials. Then, a specific amount of TMB, DA, and AzBTS solution was added to the individual catalyst solution, and the oxidation reaction was allowed to proceed for 30 minutes at room temperature. The catalyst was then recovered by centrifugation and decantation, followed by UV-vis absorption measurement and analysis of reaction byproducts. The experimental procedure for kinetic analysis is given in the SI.

### 2.3. Colorimetric Hg^2+^ ion sensing

Hg^2+^ ion sensing was performed based on its chemical affinity for the thiol functional group in glutathione (GSH). The following experimental procedure was used to detect Hg^2+^ under optimal reaction conditions. Initially, 100 µL of 0.75 mM GSH aqueous solution was pre-treated with 100 µL of Hg^2+^ aqueous solution at varying concentrations (0–500 µM) for 10 min in separate sample vials. In the meantime, CeO_2−*x*_ NSs were dispersed in a pH 4.0 citrate buffer. Then, 5 mM, 100 µL TMB solution was added to each GSH + Hg^2+^ solution, followed by the addition of 2 mL 0.25 mg per mL CeO_2−*x*_ NSs dispersion and mixing. The final reaction mixture was incubated at 25 °C for 30 min. Following the catalytic reaction, the catalyst was separated through centrifugation and decantation. The oxidized blue product in the supernatant was collected and analyzed using UV-vis absorbance spectroscopy. The standard addition method was used for real sample analysis. The real water samples were collected from the Banaras Hindu University campus. The collected water samples were filtered using a syringe filter. The sensing and recovery analysis were carried out following the procedure as discussed above.

### 2.4. Photocatalytic dye degradation

To further explore the use of CeO_2−*x*_ NSs in environmental remediation, the visible-light-driven photocatalytic dye degradation was carried out. The dye degradation experiments were conducted under a 50 W household white light lamp using 30 mL of 10 ppm aqueous methylene blue dye and 0.5 mg per mL catalyst. The dye and catalyst solution were stirred in the dark for 30 min to reach adsorption–desorption equilibrium before illumination. Aliquots of 2 mL were collected at 30 minute intervals, centrifuged to remove the catalyst, and then their UV-vis absorption spectra were recorded for analysis.

## 3. Result and discussions

### 3.1. Material characterizations

#### 3.1.1. X-ray diffraction

**Fig. 1:**
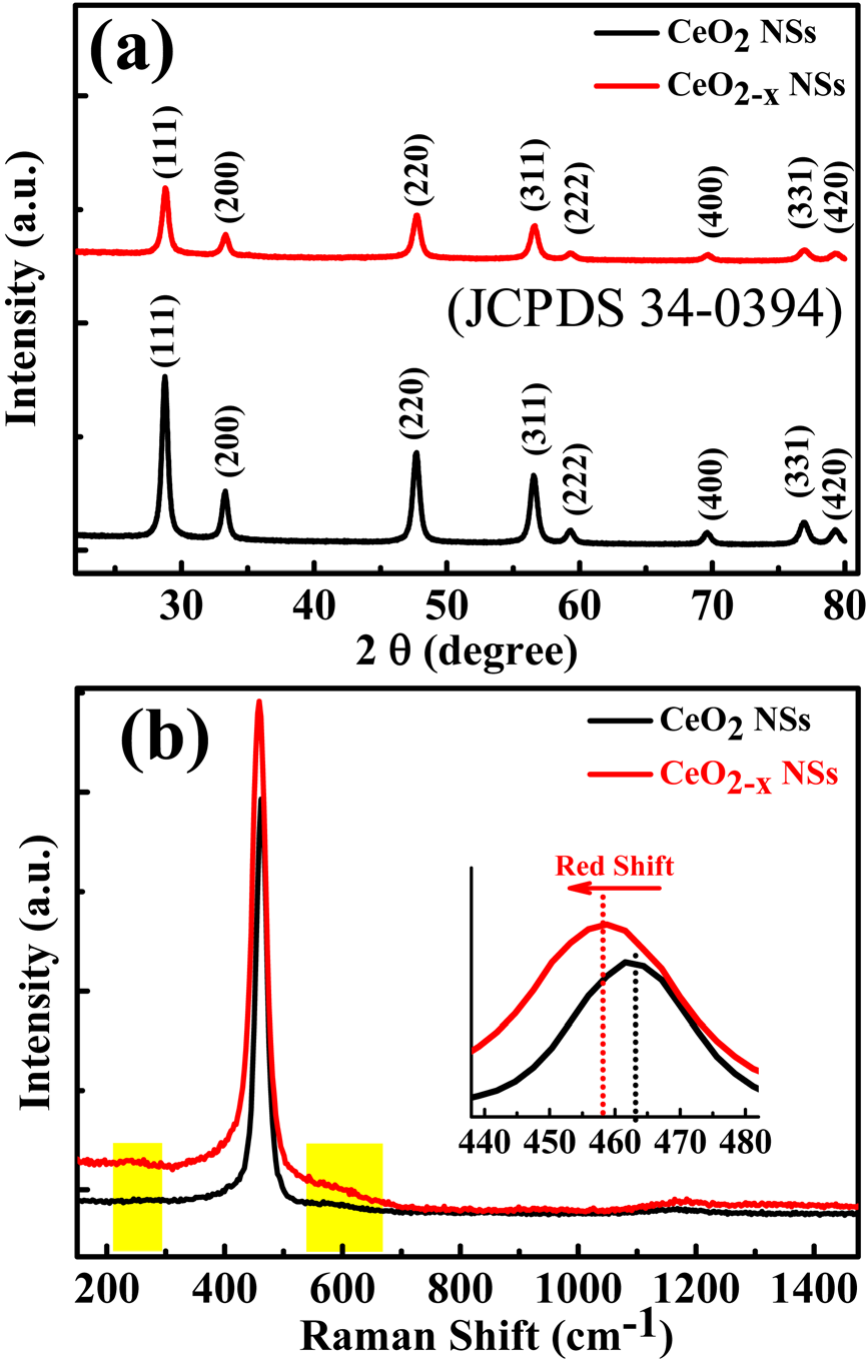
(a) XRD pattern and (b) Raman spectra of CeO_2_ NSs and CeO_2−*x*_ NSs, the inset image shows the enlarged view of the intense peak.

The crystalline properties of CeO_2_ NSs and CeO_2−*x*_ NSs were studied using the powder X-ray diffraction method, with the corresponding diffraction patterns shown in Fig. 1(a). No appreciable changes in the diffraction patterns of CeO_2_ NSs and CeO_2−*x*_ NSs were observed, confirming the stability of the crystalline properties after the reduction reaction. The diffraction peaks are centred at 2*θ* values 28.74, 33.28, 47.69, 56.55, 59.29, 69.62, 76.92, and 79.28, corresponding to the (111), (200), (220), (311), (222), (400), (331), and (420) reflection planes of face centred cubic fluorite structure of CeO_2_ (ICDD 34-0394). The lattice parameters were calculated as follows:^33^

The average crystallite size was calculated by applying the Scherrer eqn (1) to all diffraction peaks.1
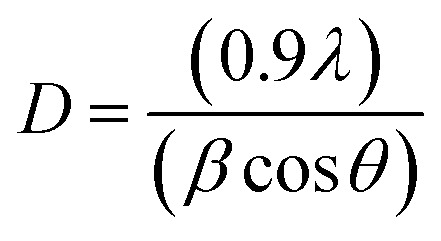


The lattice constant (*a*) was calculated using the standard cubic indexation method from the most intense (111) diffraction peak using the eqn (2)

2 *a* = *d*(*h*^2^ + *k*^2^ + *l*^2^)^2^

where (*hkl*) are the Miller indices and *d* is the interplanar spacing calculated using Bragg's eqn (3), given by

3 2*d* sin(*θ*) = *nλ*

The average crystallite sizes of CeO_2_ NSs and CeO_2−*x*_ NSs were found to be 14.27 and 13.57 nm, respectively. The lattice parameters for CeO_2_ NSs and CeO_2−*x*_ NSs were found to be 5.374 and 5.364 Å, respectively.

#### 3.1.2. Raman spectral analysis

The presence of oxygen vacancies and defects in the lattice structure of ceria causes shifts in its vibrational modes. Therefore, Raman scattering measurements were conducted to analyse the vibrational modes that can directly indicate the formation and presence of oxygen vacancies. Raman spectra of CeO_2_ NSs and CeO_2−*x*_ NSs are shown in Fig. 1(b). The most intense peak around 463 cm^−1^ for CeO_2_ NSs reflects the triply degenerate F_2g_ symmetric stretching vibrations of Ce–O bonds. A noticeable red shift was observed in this vibrational mode of CeO_2−*x*_ NSs, with the peak centred at approximately 458 cm^−1^. A shift towards lower frequencies in the F_2g_ band can be attributed to the increase in lattice volume caused by the reduction of Ce^4+^ ions to Ce^3+^ due to oxygen vacancy formation.^33^ Furthermore, an increased asymmetry and peak broadening were also observed due to the presence of oxygen vacancies and alteration in interatomic force after reduction. Additionally, two weak second-order Raman bands were observed at around 245 and 580 cm^−1^, ascribed to the transverse acoustic and longitudinal optical modes, respectively. A band around 580 cm^−1^ appeared in the spectrum of CeO_2−*x*_ NSs, which can be ascribed to the intrinsic oxygen vacancies resulting from the nonstoichiometry and the defects in the CeO_2−*x*_ NSs lattice.^34–36^

#### 3.1.3. FE-SEM and TEM analysis

**Fig. 2:**
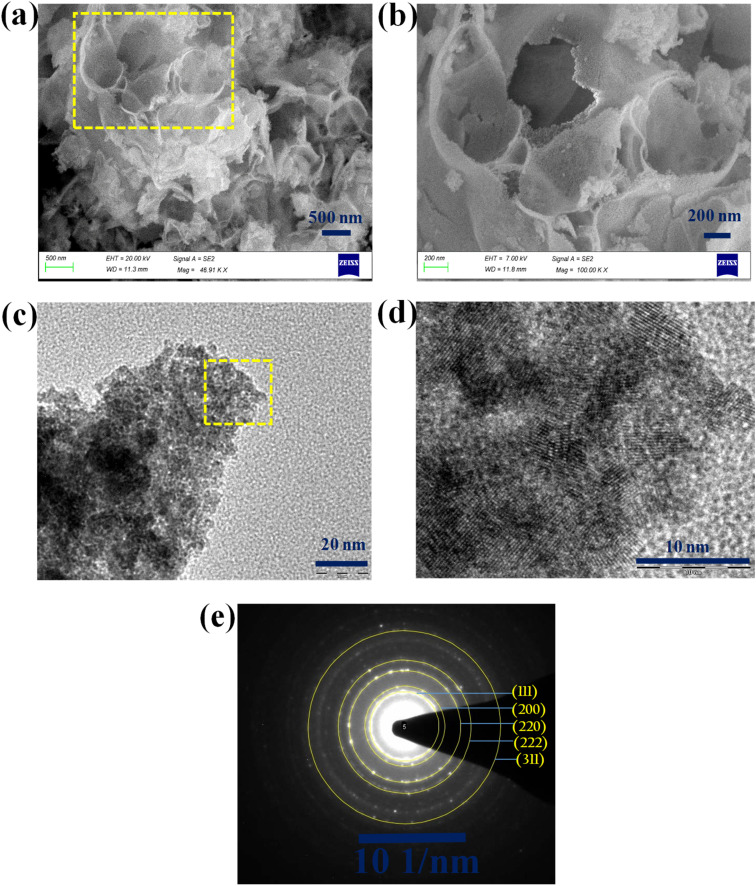
(a) low and (b) high magnification FE-SEM images, (c) TEM image (d) HR-TEM and (e) corresponding SAED pattern of CeO_2−*x*_ NSs.

Field emission-scanning electron microscopy (FE-SEM) and transmission electron microscopy (TEM) analysis were performed to visualise the morphology of CeO_2−*x*_ NSs. The FE-SEM image of CeO_2−*x*_ NSs, Fig. 2(a), reveals the formation of crumpled and wrinkled sheet-like nanostructures. The irregularly stacked nanosheets (NSs) form a three-dimensional interconnected hierarchical network. These NSs have nanometer to submicrometer lateral dimensions and a significantly reduced thickness, suggesting the formation of ultrathin NSs. The high-magnification FE-SEM image, Fig. 2(b), reveals that the surface of these NSs is rough and porous, as well as the presence of abundant interstitial voids within the three-dimensional hierarchical network. The presence of these features in CeO_2−*x*_ NSs could provide a high surface area and exposed catalytically active sites, which are advantageous for improving catalytic redox efficiency and thereby promoting nanozyme mimic activity.

The detailed morphological characterization of CeO_2_ NSs can be found in our previous report.^32^ The TEM micrograph of CeO_2−*x*_ NSs shown in Fig. 2(c) confirms the retention of 2D nanostructure after reduction with NaBH_4_. The high-resolution TEM (HR-TEM) micrograph shown in Fig. 2(d) reveals that the NSs consist of randomly oriented crystalline domains with sharp and distinguishable lattice fringes. Fig. 2(e) represents the selected area electron diffraction (SAED) pattern of CeO_2−*x*_ NSs. The SAED pattern consists of concentric diffraction rings, confirming the polycrystalline nature of CeO_2−*x*_ NSs.

#### 3.1.4. X-ray photoelectron spectral analysis

**Fig. 3:**
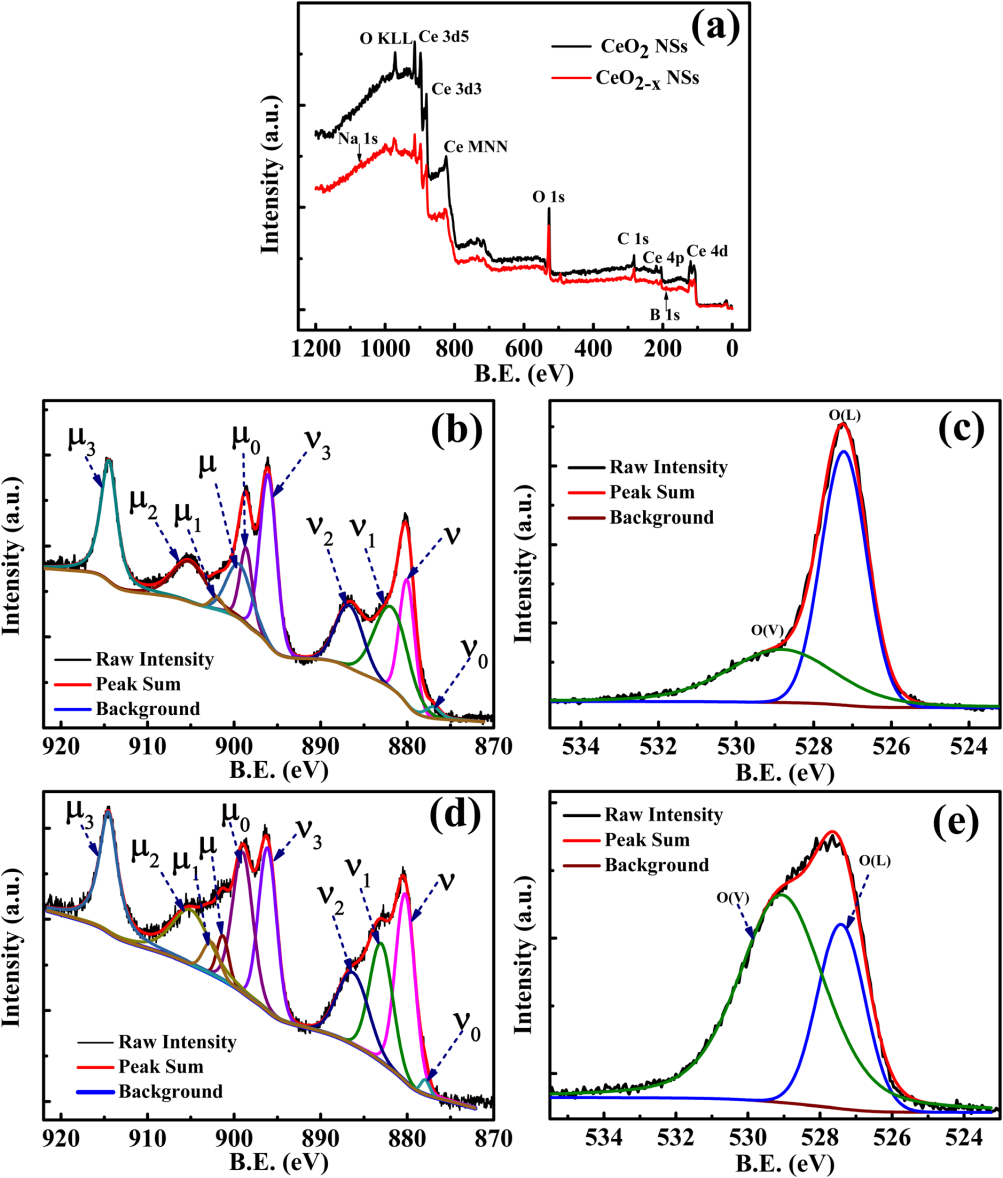
(a) Survey spectra of CeO_2_ NSs and CeO_2−*x*_ NSs; high-resolution XPS spectra of (b) Ce, (c) O corresponding to CeO_2_ NSs; and (d) Ce, (e) O corresponding to CeO_2−*x*_ NSs.

The surface elemental compositions of CeO_2_ NSs and CeO_2−*x*_ NSs were analyzed using X-ray photoelectron spectroscopy (XPS). The XPS survey spectra are shown in Fig. 3(a). In the survey spectrum of CeO_2_ NSs, Ce, O, and C were detected, while in the case of CeO_2−*x*_ NSs, Na and B were also detected in addition to Ce, O, and C, albeit with negligible intensities. The relative intensities of Na and B are considerably different, suggesting the presence of their disproportional content. This suggests that the small presence of Na and B on the surface of CeO_2−*x*_ NSs is likely due to their covalent bonding with it.^37^ The presence of a sharp C 1s spectral peak could be due to the carbonization of sugar templates during the calcination of molten syrup.

The high-resolution core-level Ce 3d XPS spectra of CeO_2_ NSs and CeO_2−*x*_ NSs are shown in Fig. 3(b) and (d), respectively. For CeO_2_ NSs, with the presence of mixed valence states Ce^3+^ and Ce^4+^, 3d core level spectra comprise 10 peaks. According to Burroughs *et al.*, the deconvoluted spectral peaks labelled as µ, µ_2_, µ_3,_ corresponding to 3d_5/2_ and ν, ν_2_, ν_3_ corresponding to 3d_3/2_, are associated with Ce^4+^, the spectral peaks labelled as µ_0_, µ_1,_ ν_0_, and ν_1_ are associated with Ce^3+^.^38^ The increased intensity of µ_0_ and ν_1_ peaks at 899 and 883 eV after reduction can be attributed to the high concentration of Ce^3+^. The atomic fraction of Ce^3+^ can be estimated based on the integral area of its respective spectral peaks from the following weight ratio4
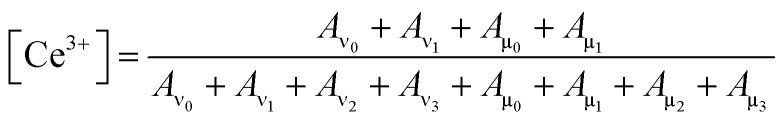
where, *A*_x_ (x = ν_0_, ν, ν_1_ ν_2_, ν_3_, µ_0_, µ, µ_1,_ µ_2_, µ_3_) represents the area under the respective spectral peaks. The calculated atomic fractions of Ce^3+^ in CeO_2_ NSs and CeO_2−*x*_ NSs were found to be 0.19 and 0.29, respectively. Thus, XPS analysis confirms that the CeO_2−*x*_ NSs possess a higher fraction of Ce^3+^, hence more defects/oxygen vacancies compared to CeO_2_ NSs. Fig. 3(c) and (e) present the core-level O 1s spectra of CeO_2_ NSs and CeO_2−*x*_ NSs, respectively. In both cases, the high-resolution spectra can be deconvoluted into two peaks. The spectral peak observed near 527 eV is attributed to the lattice oxygen atom designated as O(L), while the other peak observed near 529 eV is attributed to the adsorbed oxygen species/oxygen vacancies near the vicinity of Ce^3+^, designated as O(V). The O(V) peak for CeO_2−*x*_ NSs appeared more pronounced compared to the respective peak from CeO_2_ NSs, further confirming the formation of oxygen vacancies after reduction with NaBH_4_ in N_2_ atmosphere. The O(V) peak has a broader line profile, suggesting different coordination of oxygen in oxygen vacancy-rich CeO_2−*x*_ NSs.^39,40^ Similar weight ratio analysis for O 1s spectra shows that the O(V) fraction, related to the oxygen vacancy, for CeO_2_ NSs and CeO_2−*x*_ NSs was calculated to be 0.36 and 0.70, respectively. The surface atomic composition of CeO_2_ NSs and CeO_2−*x*_ NSs was calculated and given in Table 1. CeO_2−*x*_ NSs contain a higher Ce^3+^ atomic fraction compared to pristine CeO_2_ NSs. Since oxygen vacancies in CeO_2_ are closely associated with the reduction of Ce^4+^ to Ce^3+^, the surface oxygen vacancy concentration is higher in CeO_2−*x*_ NSs with respect to CeO_2_ NSs. The overall surface percentage of oxygen vacancy on CeO_2−*x*_ NSs are higher [O(V): (Table 1). 36.89%] compared with pristine CeO_2_ NSs [O(V): 16.04%].

**Table 1:** Surface elemental compositions of CeO_2_ NSs and CeO_2−*x*_ NS

Surface composition (atomic percentage)							
Sample/element	Ce 3d (total)	Ce 3d (Ce^3+^)	Ce 3d (Ce^4+^)	O 1s (total)	O 1s (V)	O 1s (L)	C 1s
CeO_2_ NSs	17.92	3.14	14.78	44.56	16.04	28.51	37.1
CeO_2−*x*_ NSs	9.85	2.84	7.01	52.70	36.89	15.81	37.52

#### 3.1.5. UV-vis DRS analysis

**Fig. 4:**
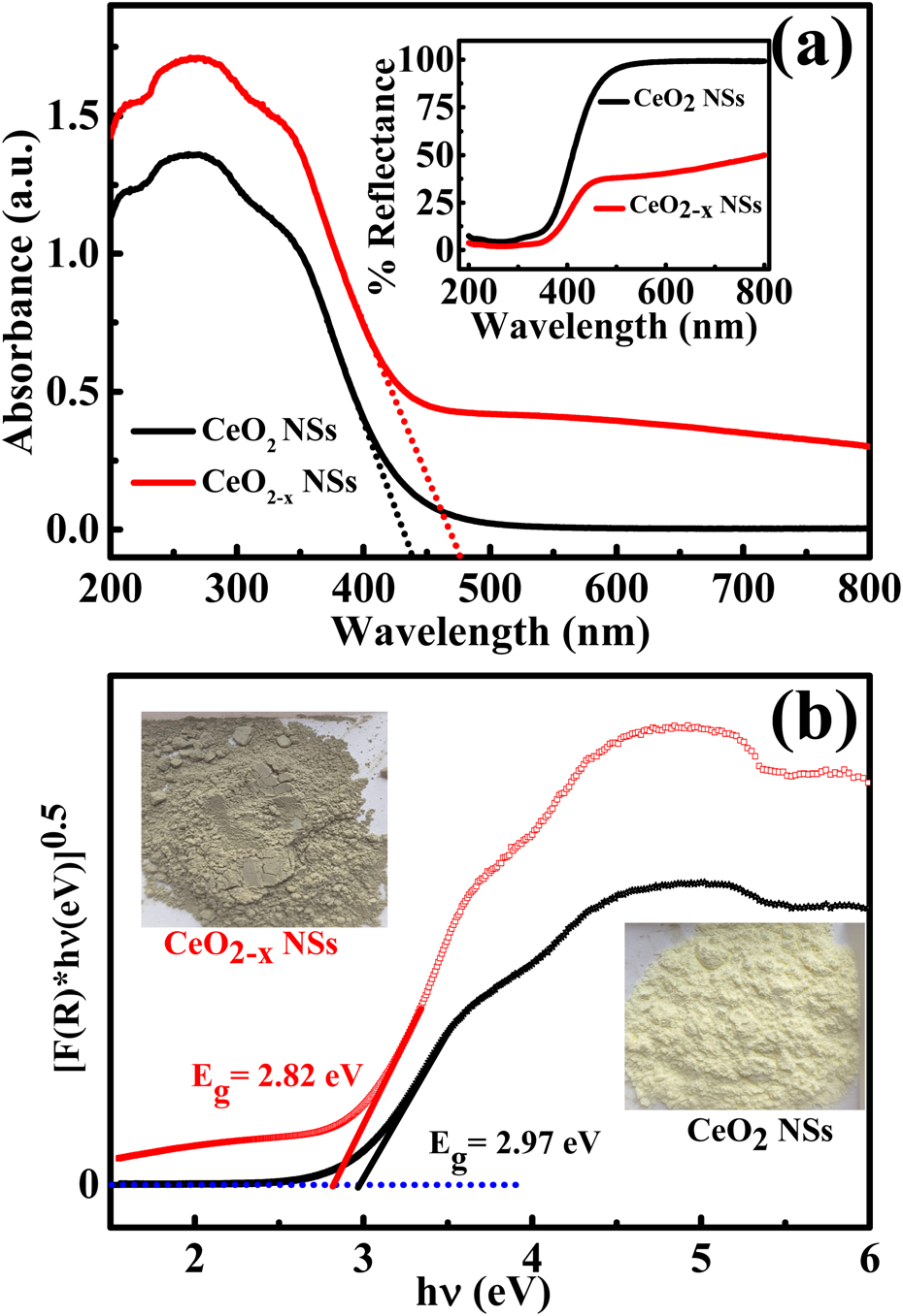
(a) UV-vis absorbance spectra (inset: UV-DRS spectra) and (b) corresponding Tauc's plots of CeO_2_ NSs and CeO_2−*x*_ NSs, the inset images show digital photographs of powder samples.

The optical properties of CeO_2_ NSs and CeO_2−*x*_ NSs were studied using diffuse reflectance spectroscopy (DRS). The UV-vis absorbance spectra and reflectance spectra are presented in Fig. 4(a). The optical absorption spectral analysis indicated that CeO_2−*x*_ NSs exhibited significantly higher absorption in the UV, visible and near-infrared region (NIR) compared to CeO_2_ NSs. As-prepared CeO_2_ NSs displayed a light-yellow hue, inset image Fig. 4(b), with an absorption onset at 435 nm. Post-reduction, the sample CeO_2−*x*_ NSs exhibited a grey appearance, inset image Fig. 4(b), with the absorption edge shifting to 465 nm along with a significant broadening of the photo-absorption profile. The red shift in the absorption can be due to the presence of oxygen vacancies.

The optical energy band gaps were estimated using Tauc's plot method, explained elsewhere in detail.^41^ In brief, the band gap energies (*E*_g_) were estimated based on the relationship between the energy-dependent Kubelka–Munk function *F*(*R*) and *E*_g_ as given by

5 [*F*(*R*) × (*ℏν*)]^1/*n*^ = *B*(*ℏν* − *E*_g_)

Accordingly, plotting [*F*(*R*) × (*ℏν*)]^1/*n*^ against *ℏν*, and extrapolating the linear portion of the plot to zero gives *E*_g_. The Tauc's plots are shown in Fig. 4(b). The band gap values were calculated to be 2.97 and 2.82 eV for CeO_2_ NSs and CeO_2−*x*_ NSs, respectively. The observed broadening in photo absorption and the reduction in the band gap can be ascribed to the presence of oxygen vacancies, as such defects have been shown to facilitate sub-band formation in oxide-based semiconductors.^37,42,43^

#### 3.1.6. EPR spectra

The EPR spectra of powder samples are shown in Fig. S6, with CeO_2−*x*_ NSs exhibiting a characteristic and relatively intense EPR signal. The EPR spectra exhibited a signal centred at 3443 G, *g* = 1.971, which may be due to the presence of Ce^3+^ paramagnetic centres formed after the reduction of Ce^4+^.

#### 3.1.7. Evaluation of oxidase mimic activity of CeO_2−*x*_ NSs

**Fig. 5:**
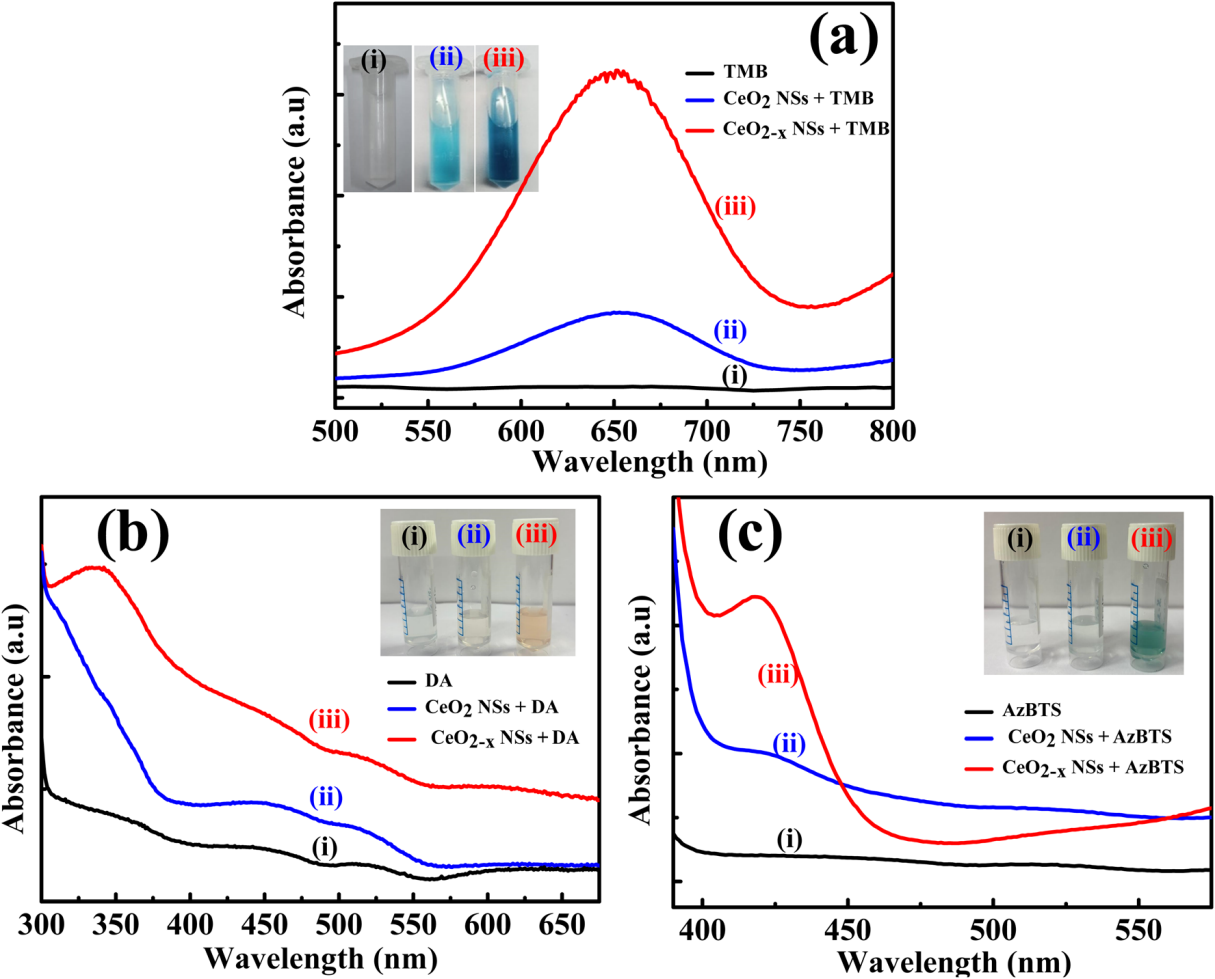
UV-vis absorption spectra (a) TMB, (b) DA and (c) AzBTS with their oxidized products, catalyzed by CeO_2_ NSs and CeO_2−*x*_ NSs, measured in citrate buffer of pH 4.0, 2 mL of 0.25 mg/mL catalysts with the addition of 100 µL of 5 mM oxidase substrates.

Detailed characterizations confirmed that the CeO_2−*x*_ NSs contain more Ce^3+^ ions, leading to more oxygen vacancies compared to CeO_2_ NSs. Therefore, the effect of increased oxygen vacancies on the catalytic performance of CeO_2_ NSs and CeO_2−*x*_ NSs was compared by assessing their intrinsic oxidase mimic activities. The oxidase–mimic activity was tested using TMB, DA, and AzBTS as the chromogenic substrates, as they become colored (blue, red and green, respectively) after oxidation in acidic conditions. The comparative catalytic efficiencies can be directly observed with the naked eye and easily quantified using a simple UV-visible spectrophotometer. Fig. 5(a) shows the UV-vis absorption spectra of TMB after oxidation in the presence of CeO_2_ NSs and CeO_2−*x*_ NSs. Pristine TMB is colorless, as shown in the inset image (i) in Fig. 5(a), and shows no absorbance spectrum. When TMB was incubated with CeO_2_ NSs and CeO_2−*x*_ NSs in an acidic buffer, a blue color developed, as shown in the inset images (ii) and (iii) in Fig. 5(a), with a characteristic absorption band centred at 652 nm. Oxidation reaction, in the presence of CeO_2−*x*_ NSs, results in a more intense blue color and higher absorbance spectra. To further assure the superiority of the oxidase activity of CeO_2−*x*_ NSs over its CeO_2_ NSs counterpart, two more chromogenic oxidase substrates, DA and AzBTS, were subjected to a similar oxidation reaction. The results are shown in Fig. 5(b) and (c), respectively. The color change and UV-vis absorption spectral analysis of oxidation reactions of DA and AzBTS, follow a similar trend to that of TMB. The results show that CeO_2−*x*_ NSs have superior catalytic activity compared to CeO_2_ NSs. These experimental results confirmed that the reduction of CeO_2_ NSs to CeO_2−*x*_ NSs significantly enhanced its intrinsic oxidase mimic activity, which can catalyse the oxidation of a range of oxidase substrates more efficiently, even without a strong oxidizing agent such as H_2_O_2_. Utilizing the superior oxidase–mimetic activity, CeO_2−*x*_ NSs were further used to develop colorimetric sensor for the detection Hg^2+^ in water samples. Quantitative spectral changes before and after oxidation of three substrates by CeO_2−*x*_ NSs is shown in Fig. S3. Based on the superiority of TMB over AzBTS and DA (discussed in detail in SI) TMB was selected as chromogenic oxidative substrate for further study.

**Fig. 6:**
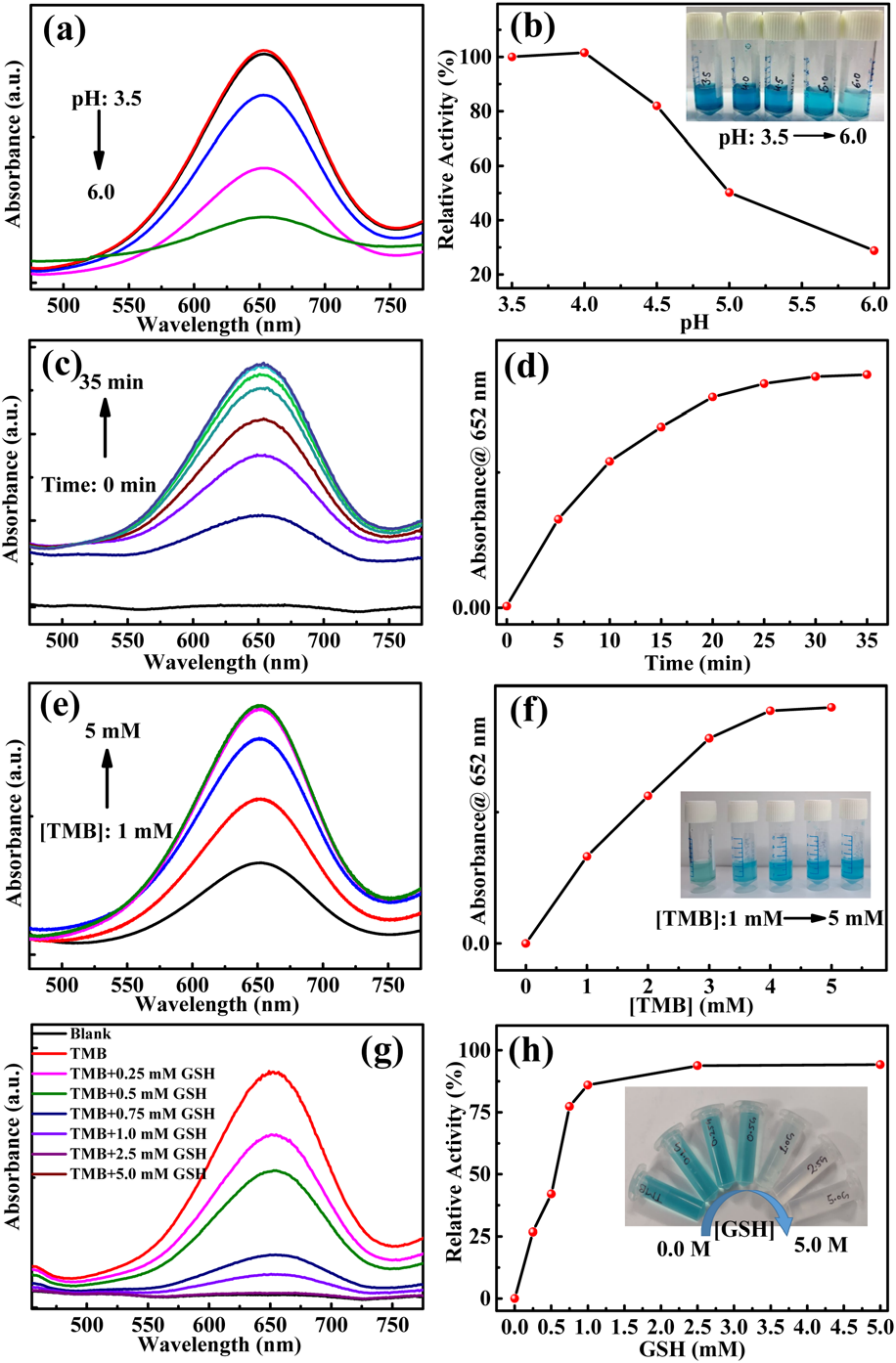
(a) UV-vis absorption spectra of ox-TMB in varying pH (0.25 mg per mL CeO_2−*x*_ NSs, 5 mM TMB) and (b) corresponding relative activity, (c) UV-vis absorption spectra of ox-TMB at different reaction time (0.25 mg per mL CeO_2−*x*_ NSs, 5 mM TMB, pH 4.0) and (d) corresponding absorbance changes, (e) UV-vis absorption spectra of ox-TMB in varying TMB concentration (0.25 mg per mL CeO_2−*x*_ NSs, pH 4.0) and (f) corresponding absorbance changes, (g) UV-vis absorption spectra of ox-TMB in varying GSH concentration (0.25 mg per mL CeO_2−*x*_ NSs, 5 mM TMB, pH 4.0) and (h) corresponding relative activity, catalyzed by CeO_2−*x*_ NSs. Digital images showing the visible color changes in each case.

Since the nanozymatic activity strongly depends on reaction conditions. Therefore, to determine the optimum reaction conditions, the effects of buffer pH, reaction time, concentration of the oxidative substrate TMB, and the concentration of GSH were assessed. Fig. 6(a) shows the UV-vis absorption spectra of ox-TMB in citric acid buffer at varying pH, keeping the concentrations of CeO_2−*x*_ NSs and TMB fixed. The results show that the relative catalytic activity, Fig. 6(b), is superior in acidic conditions and produces the maximum blue color at pH 4.0 (inset Fig. 6(b)). This pH-dependent catalytic activity of CeO_2−*x*_ NSs is in accordance with previous reports, establishing the oxidase–mimic activity of CeO_2−*x*_ NSs. Similarly, to determine the sensors' optimum response time, the absorbance was monitored over time, keeping the concentrations of CeO_2−*x*_ NSs and TMB fixed. As shown in Fig. 6(c) and (d), the absorbance increases initially up to 30 min, and no change in absorbance is observed thereafter. Therefore, 30 min was selected as the optimum reaction time for further analysis. Fig. 6(e) shows the UV-vis absorbance spectra of various concentrations of ox-TMB produced by a 0.25 mg per mL CeO_2−*x*_ NSs dispersion at pH 4.0. The results, Fig. 6(f), show that the optimum concentration of TMB is 5 mM. Based on these detailed optimization studies, citric acid buffer at pH 4.0, a 30 min reaction time, a catalyst amount of 0.25 mg mL^−1^, and 5 mM TMB were selected for further studies. Since GSH can inhibit the oxidation reaction, it is crucial to optimize the concentration of GSH as well. Fig. 6(g) shows the UV-vis absorption spectra of ox-TMB as a function of the concentration of GSH. The addition of 1 mM GSH resulted in nearly complete quenching of the reaction and disappearance of the blue color. No significant color change, Fig. 6(h), was observed thereafter. Therefore, to record a noticeable relative change in the color intensity after addition of Hg^2+^, the 0.75 mM GSH, with a relative quenching activity of ∼75%, was selected for further analysis.

The kinetics study of TMB oxidation was also performed. The TMB oxidation follows the typical Michaelis–Menten kinetics shown in Fig. S4. The details of kinetic parameters are given in the SI, and the calculated parameters are given in Table S2. The Michaelis–Menten constant (*K*_m_), related to the affinity of TMB towards the catalysts, of CeO_2−*x*_ NSs for TMB was *ca.* 65.4 µM. A lower *K*_m_ value reflects a higher affinity of the given enzyme for that substrate, and *vice versa*. This value is comparable to, or even lower than, those reported for nanoceria-based oxidase mimics and CeO_2_ NSs, indicating that CeO_2−*x*_ NSs exhibit enhanced binding affinity for TMB.

#### 3.1.8. Off–on colorimetric sensing of Hg^2+^

**Fig. 7:**
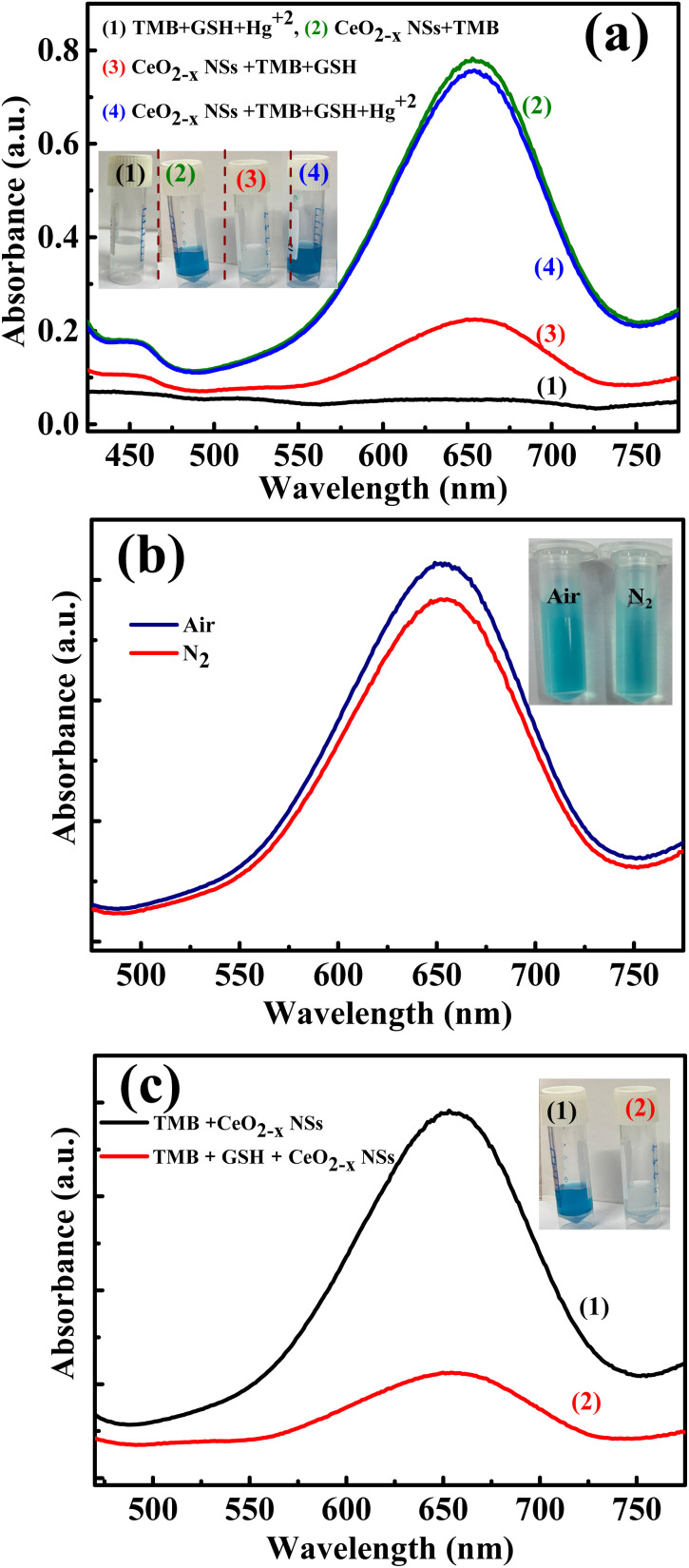
(a) UV-vis absorption spectra of (1) TMB + GSH + Hg^2+^, (2) CeO_2−*x*_ NSs + TMB, (3) CeO_2−*x*_ NSs + TMB + GSH, (4) CeO_2−*x*_ NSs + TMB + GSH + Hg^2+^, the inset images show the respective color changes, UV-vis absorbance spectra of ox-TMB in (b) air-saturated and N_2_-saturated atmospheres and (c) in absence and presence of GSH under optimum experimental conditions.

GSH can both inhibit the oxidation of TMB and reduce the blue ox-TMB to colorless TMB. Fig. 7(a) presents the absorbance spectra of ox-TMB catalyzed by CeO_2−*x*_ NSs in the absence and presence of GSH, respectively. The results show that in the absence of GSH, the green curve and inset image 2, a pronounced blue color appeared. Whereas when the reaction was conducted in the presence of GSH, the TMB oxidation was significantly inhibited and resulted in a significantly reduced color, shown in the red curve and inset image 3. This stage is the off stage. Interestingly, when Hg^2+^ was pre-incubated with GSH, the blue color was restored, as shown in the blue curve and inset image 4. This stage is on stage. Utilizing this off–on phenomenon, a fast and simple colorimetric Hg^2+^ sensor was developed.

#### 3.1.9. Sensing mechanism

It is well known that in the oxidase activity, the oxidase enzyme catalyzes the redox reactions involving the molecular reaction as an electron acceptor. Since in this reaction system no external oxidant was present, it was supposed that dissolved O_2_ played the role of oxidant. To verify the role of molecular O_2_ in the oxidation of TMB during sensing, a set of two experiments was performed under the optimum reaction conditions. First, the sensing reaction was conducted in an air-saturated environment and the second one in an N_2_-saturated environment. The UV-vis absorbance measurements, Fig. 7(b), show reduced absorbance and less intense coloration, inset Fig. 7(b), in the N_2_-saturated reactants. These measurements indicated the involvement of dissolved O_2_ in the oxidation reaction of TMB, similar to other reported nanozymes.^44–46^ The O_2_-dependent catalytic activity establishes the oxidase–mimic activity of CeO_2−*x*_ NSs. The catalytic activity observed even after bubbling with N_2_ could be attributed to chemisorbed O_2_ on the highly reactive surface of oxygen-vacancy-rich defective CeO_2−*x*_ NSs.

**Scheme 2:**
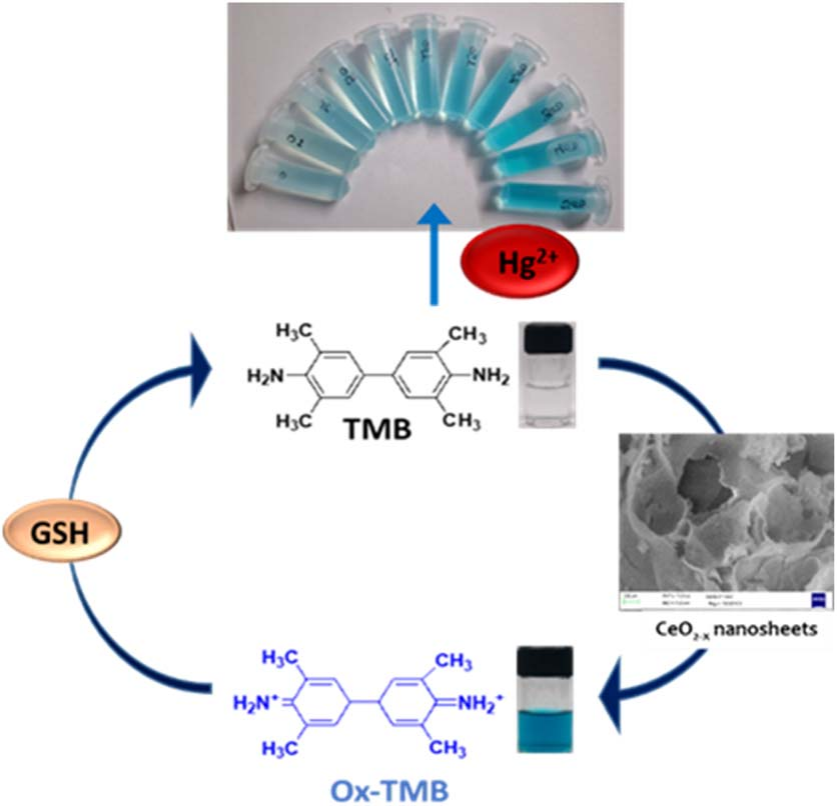
off–on reaction mechanism of Hg^2+^ sensing.

GSH, a well-known antioxidant, also acts as a scavenger of superoxide radicals (˙O_2_^−^). Therefore, to further confirm the involvement of ˙O_2_^−^ in the oxidation of TMB, this reaction was performed in the presence of GSH. As shown in Fig. 7(c), the presence of GSH significantly inhibits the oxidation reaction, resulting in much weaker coloration and lower absorbance compared to its absence. These experimental results confirm that ˙O_2_^−^ is involved in the oxidase mimetic catalytic oxidation reactions occurring on the surface of CeO_2−*x*_ NSs. Therefore, the oxidase mimetic activity of CeO_2−*x*_ NSs can be explained as shown in Scheme 2. The off–on sensing strategy has the following steps: (1) GSH, with its antioxidant nature, can inhibit the oxidation reaction and the sensor will remain in the off state. (2) Due to the thiol functionality of Hg^2+^ with GSH, the blue colored cation radicals can be restored. The involved chemical reactions are given and explained in Scheme S1 of SI. EPR and radical scavenging measurements, Fig. S5, further confirm the presence of reactive oxygen species during TMB oxidation. Species associated with O_2_ radicals play the major role in the oxidase–mimic activity of CeO_2−*x*_ NSs.

## 4. Applications

### 4.1. Hg^2+^ sensing

**Fig. 8:**
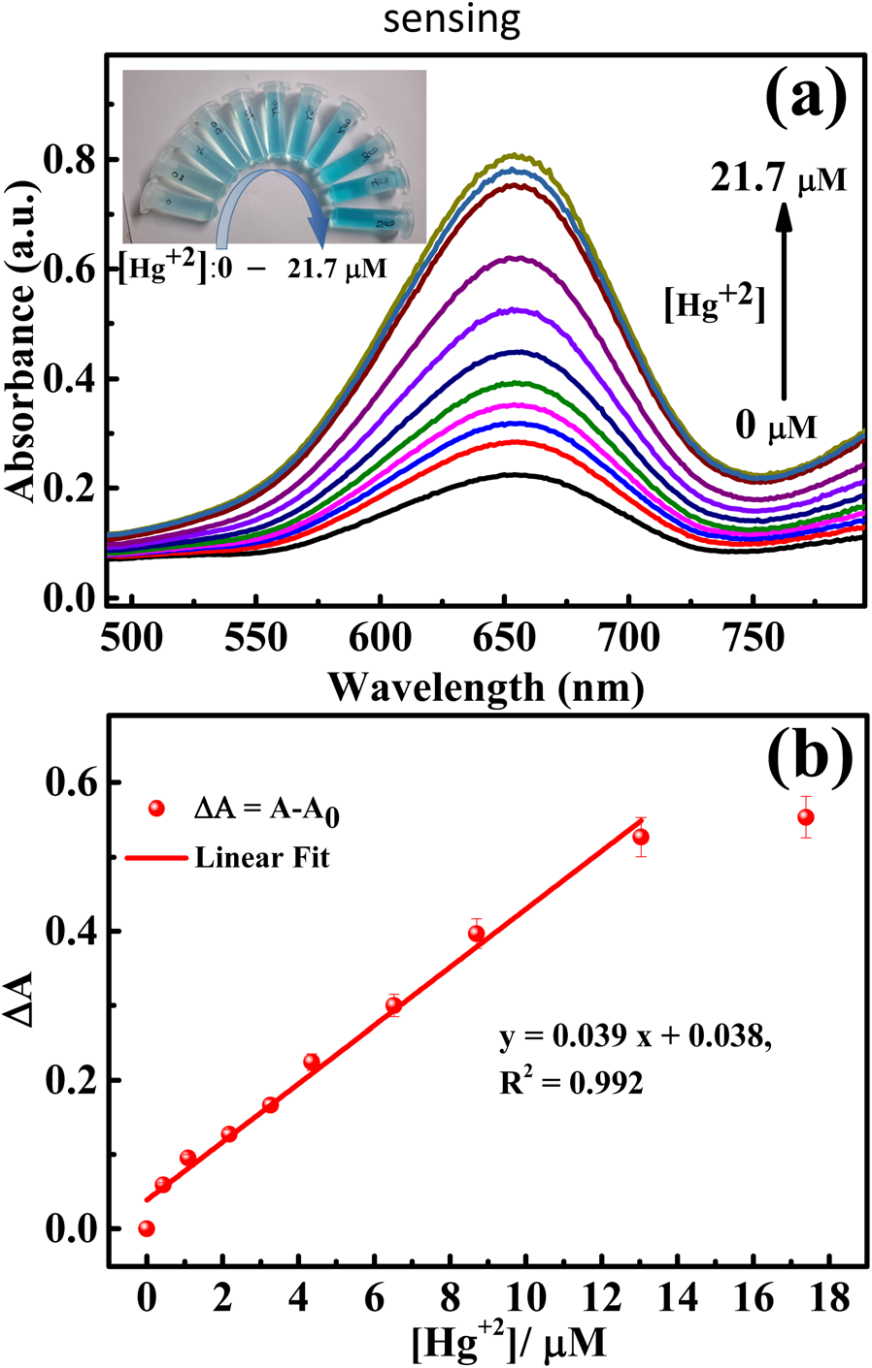
(a) UV-vis absorption spectra of ox-TMB in the presence of various [Hg^2+^], inset image show the respective color change, and (b) corresponding calibration plot.

To develop a colorimetric assay for the detection of Hg^2+^ ions, changes in colour intensity and absorbance across a range of Hg^2+^ concentrations -were investigated. As shown in Fig. 8(a), the light blue color and corresponding absorbance intensity gradually increase with the progressive rise in Hg^2+^ concentration. The calibration plot between the change in absorbance (Δ*A*) measured at 652 nm and Hg^2+^ concentration ([Hg^2+^]) is shown in Fig. 8(b). The calibration plot showed a good linear relationship (Δ*A* = 0.039[Hg^2+^] + 0.038; *R*^2^ = 0.992) between change in absorbance values and corresponding [Hg^2+^] from 0.0 µM to 17.4 µM. The limit of detection (LOD) was calculated using the relationship LOD = 3 × (*σ*/*m*), where ‘*σ*’ represents the standard deviation of 10 blank samples and ‘*m*’ is the slope of the calibration plot. The LOD for the Hg^2+^ sensing was found to be as low as 24.92 nM. A comparative study, Table S3, of the present system is made with the previously reported nanozymes. The comparative analysis demonstrates that the pristine CeO_2−*x*_ NSs exhibit comparable and, in some cases, superior detection performance relative to noble metal and hybrid nanocomposite-based oxidase mimic nanozymes. Furthermore, unlike the conventional CeO_2_ nanoparticles or their composite nanozymes, 2D CeO_2−*x*_ NSs show promising H_2_O_2_ free nanozymatic activity for sensing applications owing to oxygen-vacancy-rich, enhanced surface accessibility, Fig. S1, abundant exposed active sites, and improved charge-transfer characteristics.

### 4.2. Selectivity, interference, real sample test, reusability and stability analysis

**Fig. 9:**
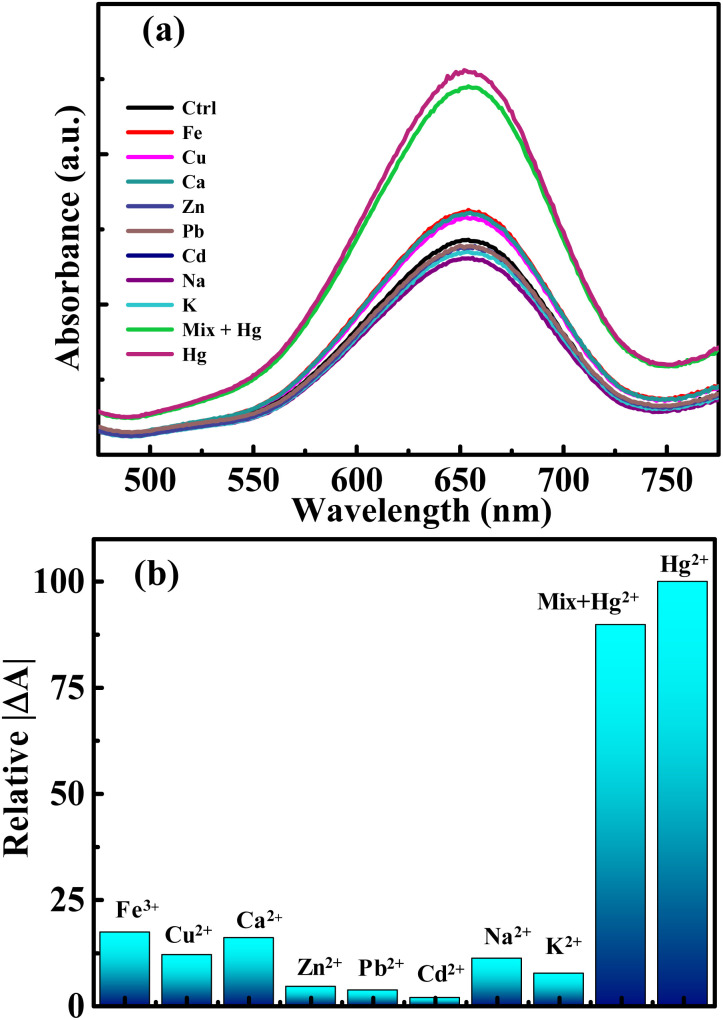
(a) UV-vis absorption spectra of ox-TMB recorded in the presence of different metal ions, a mixture of them with Hg^2+^, and Hg^2+^; a bar graph in (b) shows the corresponding Δ*A* responses.

To explore the selectivity of Hg^2+^ ion detection, the sensing experiments were performed in the presence of various other potential interfering metal ions as well as in a complex water sample, Fig. 9. The results show that the developed sensor is selective towards Hg^2+^. The interference data show that the coexistence of other ions does not significantly affect the response towards the Hg^2+^ sensing, underscoring the high selectivity of the GSH + TMB + CeO_2−*x*_ NSs system. Therefore, these results indicate that the proposed system is suitable for the selective sensing of Hg^2+^ in real samples. The reusability analysis of CeO_2−*x*_ NSs was evaluated by three repeated cycles, Fig. S7(a) and (b). The results show that the catalyst retains 94% activity after three consecutive cycles. Furthermore, the XRD and XPS analysis, Fig. S7(c) and (d), of pristine and recovered catalysts show no appreciable changes, confirming the stability of the catalyst after the reaction.

To demonstrate the practical application of the proposed sensing methodology, Hg^2+^ was quantified in real samples under optimum reaction conditions. The real sample analysis data is summarized in Table S4. The obtained recovery values between 91% and 111% demonstrate the reliability and practical application of the present simple and rapid colorimetric approach for Hg^2+^ sensing. At present, the CeO_2−*x*_ NSs-based colorimetric sensing platform demonstrates optimal performance in soft natural water systems, while its applicability to more complex matrices requires further validation.

In addition to the nanozymatic activity, the comparative photocatalytic efficiencies of CeO_2_ NSs and CeO_2−*x*_ NSs were also assessed by evaluating the degradation of methylene blue dye under visible light irradiation. The degradation efficiency (*η*) and degradation rate constant (*k*) were calculated from the following equations (6) and (7), respectively:6
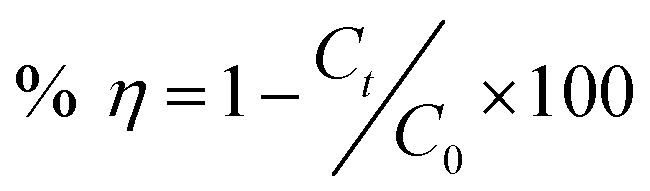
7
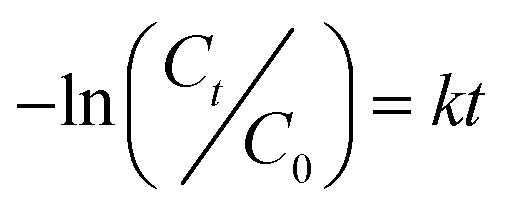


**Fig. 10:**
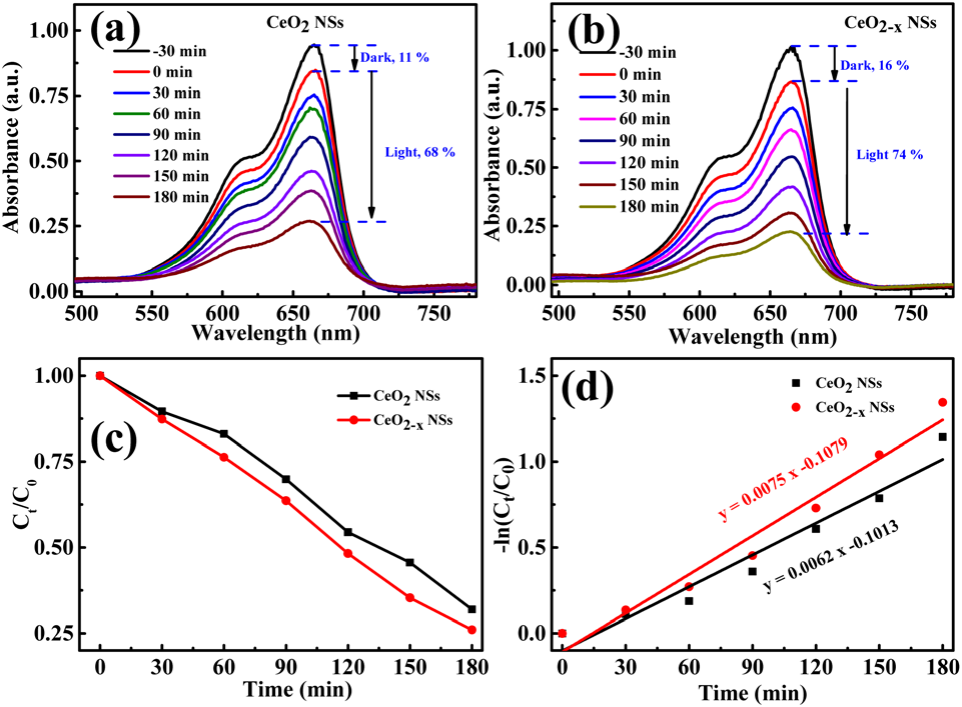
UV-vis absorption spectra of MB degradation by (a) CeO_2_ NSs and (b) CeO_2−*x*_ NSs recorded in dark conditions and after a regular interval of light illumination. Graphs (c) and (d) compare the rate and kinetics of photocatalytic dye degradation.

Fig. 10(a) and (b) show UV-vis absorption spectra of methylene blue degradation over time. Fig. 10(c) and (d) show the degradation rate and kinetic plots, resepectively. The photocatalytic degradation efficiencies/rate constants of CeO_2_ NSs and CeO_2−*x*_ NSs were found to be 68%/0.0062 min^−1^ and 74%/0.0075 min^−1^ for CeO_2_ NSs and CeO_2−*x*_ NSs, respectively, in 180 min, accounting for a 6% increment in degradation efficiency and a 13% increment in rate constant. The improved photocatalytic activity of CeO_2−*x*_ NSs could be attributed to their synergistic effects of increased accessibility to active sites with preserved porous structure, reduced band gap, improved exciton (e^−^–h^+^ pair) separation efficiency (discussed in the PL analysis (Fig. S2)) and the enhanced visible light absorption in the visible region. The developed CeO_2−*x*_ NSs exhibit dual functionality by integrating sensitive colorimetric Hg^2+^ sensing with visible light-driven photocatalytic dye degradation capability within a single catalyst system, which has rarely been reported, Table S5, for CeO_2_ based oxidase nanozymes.

## 5. Conclusions

The present work introduces a scalable and cost-effective method for synthesising novel metal-free, porous, and Oxygen-vacancy-rich 2D CeO_2−*x*_ NSs nanozyme by reducing CeO_2_ NSs with NaBH_4_ in an N_2_ atmosphere at 400 °C. Raman and XPS analyses confirm the reduction of Ce^4+^ to Ce^3+^ on the surface of CeO_2_ NSs and the creation of oxygen vacancies. The presence of oxygen vacancies in the synthesised CeO_2−*x*_ NSs significantly enhances oxidase–mimic activity, which pristine CeO_2_ NSs cannot achieve. In addition, the reduction decreases the band gap, extends light absorption into the visible range, and improves exciton separation efficiency, thereby enhancing photocatalytic activity. In addition to this, the precise regulation of oxygen vacancy concentration through controlled modulation of synthesis parameters, along with a quantitative understanding of the defect–activity relationship, needs further investigation.

Based on the intrinsic and superior oxidase–mimic activity of CeO_2−*x*_ NSs, a facile, rapid, and highly sensitive off–on colorimetric sensing method was developed for selective detection of Hg^2+^ ions. The off–on colorimetric sensing approach is based on the recovery of the colourless TMB molecule to blue-coloured TMB cation radicals, making the whole sensing approach visible to the naked eye. The developed sensor exhibits good linearity, a low detection limit, and satisfactory recovery results. Consequently, this novel “off–on” colorimetric strategy provides a reliable and effective platform for monitoring trace levels of Hg^2+^ in water samples, and visible light active photocatalytic dye degradation capability demonstrates the potential applications of CeO_2−*x*_ NSs in environmental remediation.

## Author contributions

Ashish Kumar: investigation, data curation, visualization, software, and writing – original draft. Suchi Smita Singh: investigation, resources. Suverna Trivedi: resources, writing – review & editing. Debanjan Guin: conceptualization, investigation, validation, supervision, writing – original draft. Chandra Shekhar Pati Tripathi: conceptualization, resources, validation, writing – review & editing.

## Conflicts of interest

The authors declare that they have no known competing financial interests or personal relationships that could have appeared to influence the work reported in this paper.

## Supplementary Material

RA-OLF-D6RA02432G-s001

## Data Availability

The authors declare that the data supporting this article are available from the corresponding author upon reasonable request. Supplementary information (SI) is available. See DOI: https://doi.org/10.1039/d6ra02432g.
