# Supplementary material for: Defect-rich CeO2−x nanosheets as efficient oxidase nanozymes for colorimetric Hg2+ detection and visible-light photocatalysis
Source: RSC Adv. 2026 Jul 2;16(34):33062–75. doi: 10.1039/d6ra02432g (PMC13326585; doi:10.1039/d6ra02432g)
Supplement: RA-016-D6RA02432G-s001 [file RA-016-D6RA02432G-s001.pdf]

Supplementary Information (SI) for RSC Advances.
This journal is © The Royal Society of Chemistry 2026

Supporting Information

Defect-Rich CeO_{2-x} Nanosheets as Efficient Oxidase Nanozymes for Colorimetric Hg^{2+} Detection and Visible-Light Photocatalysis

Ashish Kumar[a], Suchi Smita Singh[b], Amit Pathak[a], Suverna Trivedi [c, *], Debanjan Guin[b, *], and Chandra Shekhar Pati Tripathi[a,*]

[a]*Department of Physics, Institute of Science, Banaras Hindu University, Varanasi-221005 Uttar Pradesh, India*
[b]*Department of Chemistry, Institute of Science, Banaras Hindu University, Varanasi-221005, Uttar Pradesh, India*
[c]*Department of Chemical Engineering, Indian Institute of Technology Kharagpur, Kharagpur 721302, India*
E-mail id: strivedi@che.iitkgp.ac.in *(S.T.),* debanjan.chem@bhu.ac.in (D.G.) tripathi.csp@bhu.ac.in *(CSPT)*

Materials: Cerium (III) nitrate hexahydrate ($Ce(NO_3)_3.6H_2O$) and 2,2′-Azino-bis (3-ethylbenzothiazoline-6-sulphonic acid) diammonium salt (AzBTS) were purchased from Loba Chemie. D-glucose was provided by Fisher Scientific. 3, 3′, 5,5,5′- tetramethylbenzidine (TMB) was purchased from SRL Chemicals. Dopamine (DA) hydrochloride was purchased from Acros Organics. Sodium citrate dihydrate ($Na_3C_6H_5O_7{\cdot}2H_2O$), citric acid ($C_6H_8O_7$), absolute ethanol ($C_6H_5OH$), Sodium borohydride ($NaBH_4$), and Mercuric chloride ($HgCl_2$) were purchased from Merck. Glutathione (GSH) was purchased from BLD Pharma. All chemicals were of analytical grade and used without further purification. The deionized water (DW) was used as a solvent in synthesis and sensing experiments, otherwise specifically mentioned.

Instruments: The following analytical methods and instruments were used to characterize CeO_2 NSs and CeO_{2-x} NSs. X-ray diffraction (XRD) was performed on powder samples using a PANalytical Empyrean X-ray diffractometer. High-Resolution field emission scanning electron microscope (FE-SEM), Model: GEMINI 560 from Carl Zeiss, and A transmission electron microscope (TEM), model Tecnai G2 TWIN from FEI Company (USA), were used to image the CeO_{2-x} NSs. Surface elemental compositions were analyzed with an X-ray photoelectron spectrophotometer, model PHI 5000 VersaProbe III from Physical Electronics. Lattice phonon modes were analyzed with a Renishaw inVia Raman spectrophotometer. Diffuse reflectance spectra were obtained using a Carry 5000 UV-Vis-NIR spectrophotometer. UV-Visible absorption spectra were recorded with a UV-170 spectrophotometer from Shimadzu. N_2 adsorption-desorption analysis for surface area measurements was performed on Autosorb iQ2 from Quantachrome Instruments (USA). EPR measurements were conducted on a Bruker ELEXSYS 580 pulsed Electron Paramagnetic Resonance spectrometer.

BET Analysis: The surface area and porosity of the synthesised materials were studied using N_2 adsorption-desorption. The adsorption-desorption isotherms are shown in Figure S1. The observed hysteresis loops resemble the H_3 type according to IUPAC nomenclature, which is typically associated with the plate-like structure.

The surface area, pore size, and pore volume of CeO_2 NSs and CeO_{2-x} NSs are summarized in Table S1. The reduction treatment caused only a marginal decrease in the specific surface area from 26.855 to 26.043 $m^2\ g^{-1}$, while the pore diameter remained nearly unchanged, indicating preservation of the porous nanostructure after reduction. Therefore, the enhanced oxidase-like and photocatalytic activities of CeO_{2-x} NSs cannot be attributed solely to surface area changes, but are primarily associated with the generation of oxygen vacancies during the reduction process. These oxygen vacancies can act as electron-trapping and oxygen-activation centres, facilitating charge separation, molecular oxygen adsorption, and reactive oxygen species generation. Notably, the pore volume increased from 0.053 to 0.076 $cm^3\ g^{-1}$, which could improve reactant diffusion and accessibility to active sites. Consequently, the synergistic effect of preserved porous structure with increased accessibility to active sites and the formation of oxygen vacancies contributes to the superior catalytic performance of CeO_{2-x}

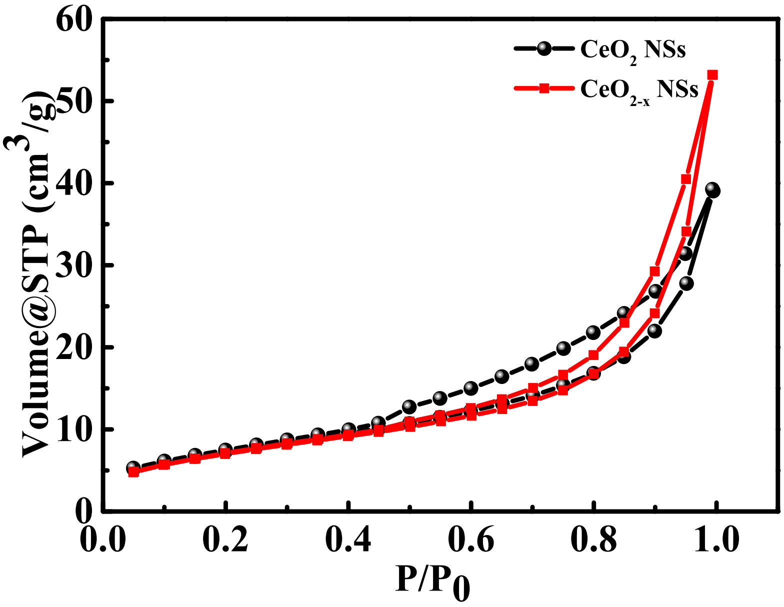

NSs.

Figure S1: N_2 Adsorption-desorption isotherms for CeO_2 NSs and CeO_{2-x} NSs

Table S1: Surface area and porous properties of CeO_2 NSs and CeO_{2-x} NSs

Sample	Surface area (m^2/g)	Pore diameter (nm)	Pore volume (cm^3/g)
**CeO_2 NSs**	26.855	4.302	0.053
**CeO_{2-x} NSs**	26.043	4.320	0.076

Photoluminescence study: Figure S2 shows the PL spectra of CeO_2 NSs and CeO_{2-x} NSs at an excitation wavelength of 300 nm. The emission peak in the UV-region originates from the electronic transitions from the Ce 4f level to O 2p level, whereas the PL emission in the visible region is attributed to the defect states related to oxyzen vacancies. The PL intensity of CeO_{2-x} NSs was markedly decreased compared to that of CeO_2 NSs. Bulk defects are generally regarded as radiative recombination centres for charge carriers, while the surface defects act

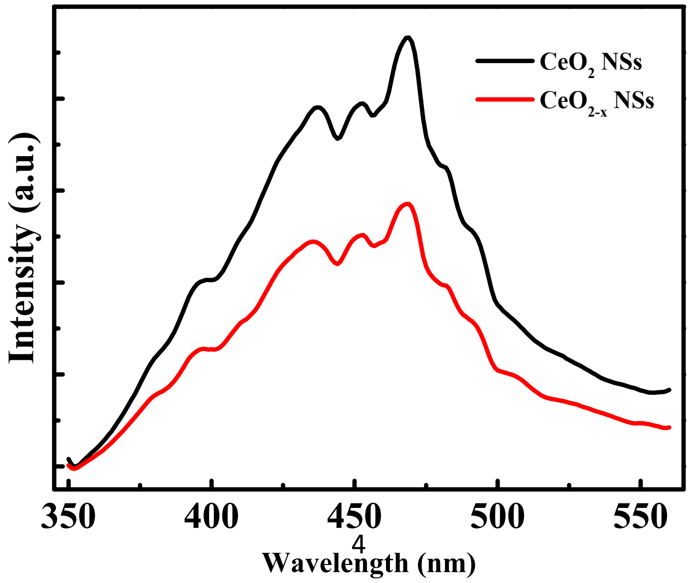

as non-radiative traps. Therefore, the decreased PL intensity after reduction suggests an enhancement in the density of surface defects.[1, 2]

Figure S2: PL spectra of CeO_2 NSs and CeO_{2-x} NSs

Selection of the most suitable Chromogenic substrate: CeO_{2-x} NSs exhibits the capability of oxidizing TMB, AzBTS and DA. Among the three chromogenic substrates TMB, DA, and AzBTS; TMB was selected for Hg^{2+} detection based on its superior optical response. The quantitative spectral changes before and after oxidation of three substrates by CeO_{2-x} NSs are compared in bar graph (d) Figure S3. The UV–Vis spectra of all substrates, recorded in both oxidised and non-oxidised states, reveal that TMB exhibits the most pronounced spectral change upon oxidation, accompanied by a distinct and intense color transition. In comparison, AzBTS shows a moderate response, while DA displays relatively weaker changes.

Also, existing literature on the measurements of molar absorption coefficient and kinetic analysis confirms that TMB is more sensitive due to its higher molar absorption coefficient and binding affinity compared to AzBTS and DA. In addition, DA, a well-known neurotransmitter, is often used as an analyte itself rather than as a chromogenic substrate. Moreover, it is highly susceptible to side reactions and interference when applied to biological samples. Therefore, to achieve a more sensitive and visually more distinguishable colorimetric effect, TMB was chosen for subsequent investigations.

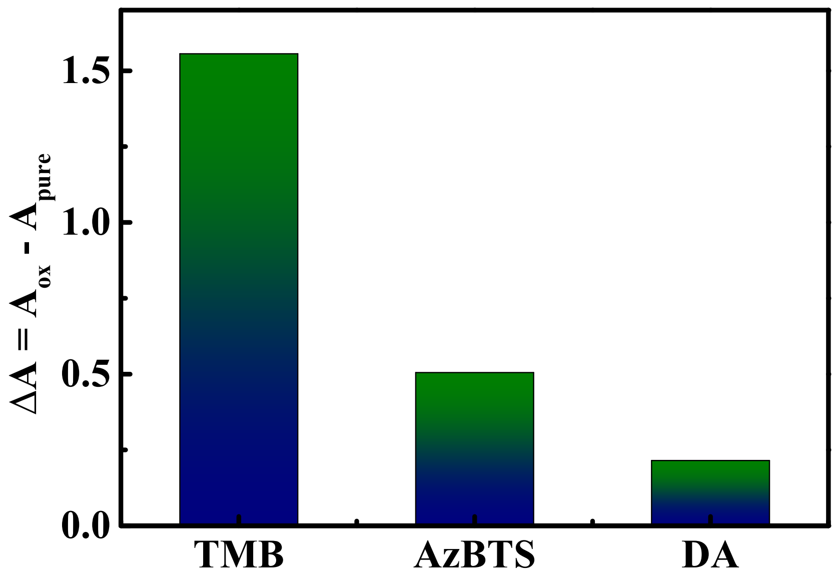

Figure S3: Quantitative spectral changes before and after oxidation of three substrates by CeO_{2-x} NSs comparing their Oxidase activity

Oxidase-mimic activity: Kinetic study

To evaluate the relative performance of the oxidase-mimic activity of CeO_{2-x} NSs, kinetic analysis was performed. The reaction kinetics were studied by noting the absorbance change at 655 nm in a time course mode. In brief, catalytic oxidation of a series of TMB concentrations in different sample vails were performed in optimum experimental conditions. Following the catalytic reaction, the aliquots from each sample vial were collected the catalyst was separated through centrifugation and decantation. The oxidized blue product in the supernatant was collected and analyzed with UV-vis absorbance spectroscopy. The kinetics of TMB oxidation were studied using Michaelis-Menten kinetics, and the reaction parameters were calculated using Lineweaver- Burk double reciprocal plot. The Michaelis-Menten and Lineweaver-Burk equations are given by

$$V = V_{max}\frac{[S]}{k_m + [S]} \qquad eq. 1$$

$$\frac{1}{V} = \frac{K_m}{V_{max}} * [S] + \frac{1}{V_{max}} \qquad eq. 2$$

Where V, and V_{max} represent the initial and maximal reaction rates, respectively, [S] represents the concentration of substrate, and k_m is the Michaelis constant. The kinetic parameters were derived by calculating the intercept ($\frac{1}{V_{max}}$) and slope ($\frac{K_m}{V_{max}}$) of the Lineweaver-Burk plot and summarised in Table S2. The obtained K_m of CeO_{2-x} for TMB was ca. 65.4 µM. This value is comparable to, or even lower than, those reported for nanoceria-based oxidase mimics and CeO_2 NSs, indicating an enhanced binding affinity of CeO_{2-x} for TMB.

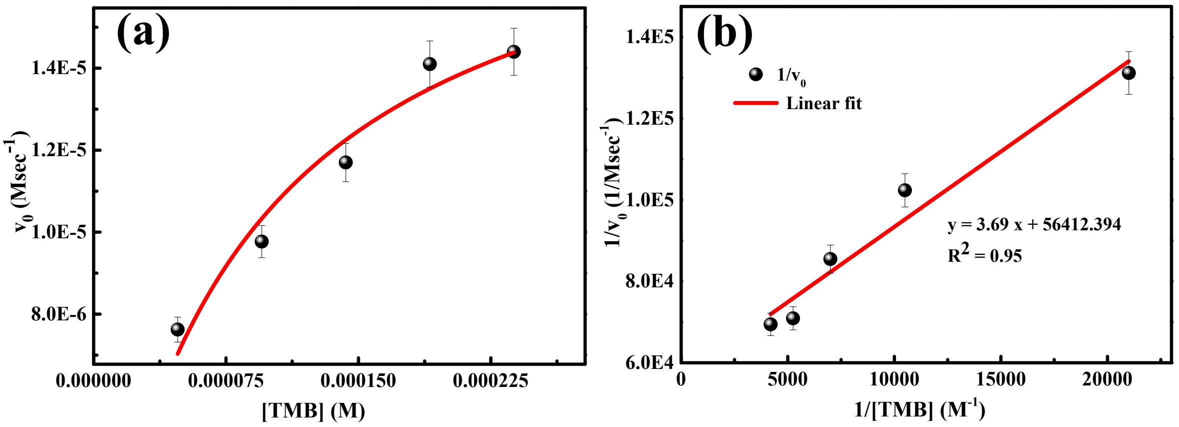

Figure S4: (a) Michaelis-Menten plot, (b) Lineweaver-Burk double reciprocal plot of CeO_{2-x} NSs

Table S2: Kinetic parameters

**Enzyme mimics**	**V_{max} ×10^{-5} (M sec^{-1})**	**K_m (μM)**	**Reference**
CeO_2-x microspheres	3.33	264	[3]
Polymer-Coated CeO_2 NPs	70	3800	[4]
CeO_2 NPs	0.010	420	[5]
CeO_2 NSs	3.40	817	[6]
CeO_{2-x} NSs	1.77	65.4	This work

Comparative study to other reported catalysts: As summarized in Table S3, the literature survey shows that many reported colorimetric detection systems rely on the use of noble metals, complex hybrid nanostructures or H_2O_2-assisted peroxidase mechanisms for Hg^{2+} sensing. The H_2O_2 -free catalytic mechanism can minimise interference associated with peroxide instability, a noble metal-free single-component catalyst can reduce the overall cost and avoid complex synthesis steps, thereby improving the operational simplicity and enhancing suitability for practical sensing applications. Furthermore, unlike conventional CeO_2 nanoparticles or its composite nanozymes, the present work employs oxygen-vacancy-rich two-dimensional CeO_{2-x} nanosheets, which provide enhanced surface accessibility, abundant exposed active sites, and improved charge-transfer characteristics. In addition to that, the developed CeO_{2-x} NSs exhibit dual functionality by integrating sensitive colorimetric Hg^{2+} sensing with visible light-driven photocatalytic dye degradation capability within a single catalyst system, which has rarely been reported for CeO_2-based oxidase nanozymes, Table S5. Therefore, the synergistic combination of defect-engineered 2D morphology, H_2O_2-free oxidase-mimic activity, and multifunctional environmental applicability highlights the novelty and significance of the present work.

Table S3: Comparison of CeO_{2-x} NSs with Reported Nanozymes for Hg^{+2} Detection and

Catalyst	Colorimetric Sensing			Photoctalytic degradation (%)	Reference
	Method	Linear range (μM)	LOD (nM)		
Ag Nanoparticles	Colorimetric	Piecewise linear 50–450	---	--	7
Ag Nanoprism	Colorimetric	0.01-0.5	3.3	--	8
cysteine-modified Au-Ag coreshell nanorods	Colorimetric	1 – 60	273	--	9
Platinum nanoparticles	Colorimetric (Peroxidase using H_2O_2)	0.01 to 0.04	5.1	--	10
g-C_3N_4/CeO_2	Colorimetric (Peroxidase using H_2O_2)	0.05 – 0.8	0.23	--	11
Porus CeO_2 Nanorods	Colorimetric (Peroxidase using H_2O_2)	0.005 – 0.100	0.31	--	12
Bimetallic Fe_3O_4@Co_3O_4/CN	Colorimetric (Peroxidase using H_2O_2)	0.1–15	17	--	13
CuO/Au@$Cu_3(BTC)_2$	Colorimetric (Oxidase without H_2O_2)	0.05 – 25	9.7	--	14
$CoSe_2$ NSs	Colorimetric (Oxidase without H_2O_2)	0.2 - 6	135	--	15
Ag@FeOOH-GO	Colorimetric (Oxidase without H_2O_2)	5 – 30	5400	--	16
Pd-MOF@Graphene NSs	Colorimetric (Oxidase without H_2O_2)	0.045 - 250	12	--	17
NH_2-MIL-101(Fe)@Cu/CeO_2 (organic-inorganic hybrid nanozyme)	Colorimetric (Peroxidase using H_2O_2)	0.1 - 4	0.7	--	18
CeO_{2-x} NSs	Colorimetric (Oxidase without H_2O_2)	1 – 17.4	24.92	74	This Work

Photocatalytic dye degradation

Table S4: Real sample analysis data of Hg^{+2} sensing in two water samples

Sample	Initial concentration (μM)	Added concentration (μM)	Found concentration (μM)	Recovery (%)
**Tap water**	Not detected	3.26<br>4.35<br>8.69	2.98<br>4.43<br>8.75	91.44<br>101.79<br>100.59
**Pond water**	Not detected	3.26<br>4.35<br>8.69	3.25<br>4.84<br>9.33	99.69<br>111.34<br>107..35

Table S5: Comparison of Photocatalytic properties of CeO_{2-x} NSs with other Reported CeO_{2-} based photocatalysts

Catalyst	Oxidase-based Colorimetric Sensing	Photo-catalytic properties					Reference
		Light Source	Dye	Time (Minute)	Degradation efficiency (%)	Rate Constant ($Minute^{-1}$)	
Irregularly Shaped CeO_2 Nanoparticles	--	UV	Malachite green	60	97.4	0.060	[19]
CeO_2/Y_2O_3 nanorods	--	UV	RhB	150	94	--	[20]
CeO_2/Al_2O_3	--	UV	MB	120	93	0.012 min−1	[20]
CeO_2 Aggregates	--	Visible	MB (10 mL 5 PPM, 25 mg catalyst)	90	44	0.0039	[21]
CeO_2 NSs<br>$CeO2_{-x}$ NSs	24 nM (LOD) for Hg	Visible	MB (30 mL 10 PPM, 15 mg catalyst)	180	68<br>74	0.0062<br>0.0074	This Work

Off-on Sensing mechanism: The chemical reaction involved in the Hg^{+2} sensing are summarised below

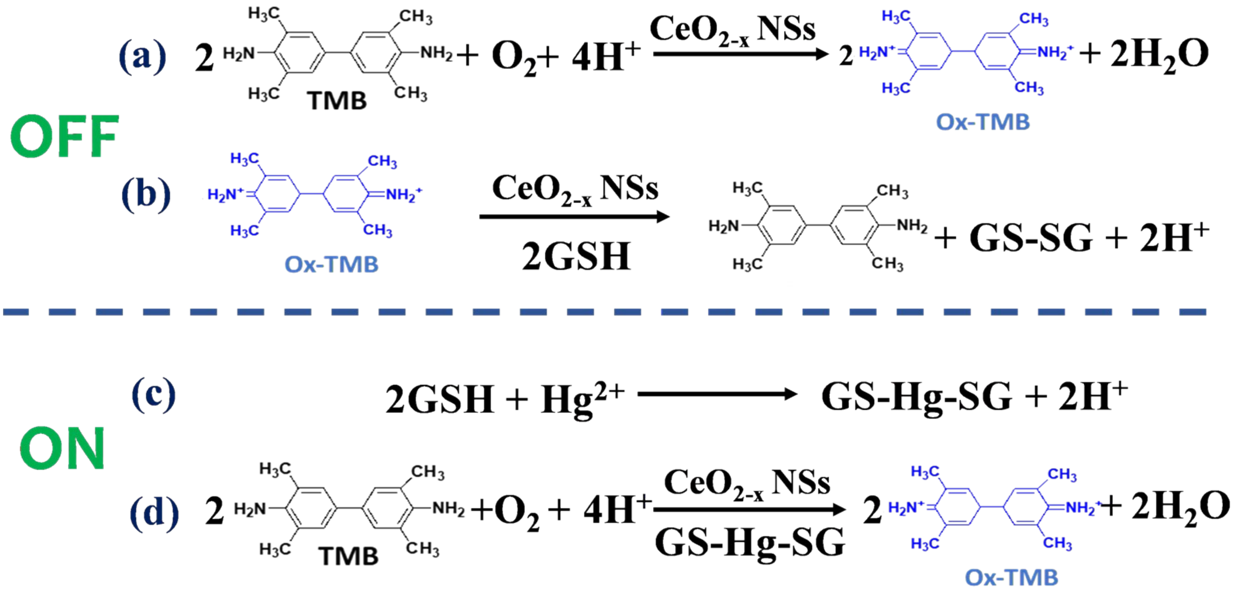

Where

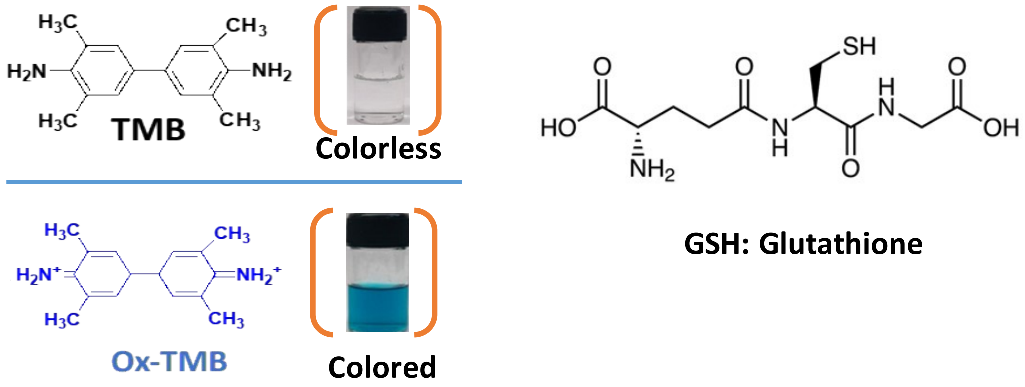

Scheme S1: *Off-On* reaction mechanism of Hg^{+2} sensing

Reaction Steps:

(a) Shows the oxidation reaction of TMB in the presence of CeO_{2-x} NSs, resulting in a colored solution.

(b) GSH can both inhibit the oxidation of TMB (no coloration) as well as reduce the blue ox-TMB to colorless TMB, leading the sensor to remain in the off state.

(c) When GSH was pretreated with Hg, it forms a complex

(d) Now, when TMB oxidation was carried out in the presence of this complex, depending on the concentration of Hg, the blue color will reappear, transitioning the sensor into the ON state

EPR and Scavenger Test: We have performed the EPR measurements to confirm the presences of reactive oxygen species during TMB oxidation. The results are given below. The DMPO spin-trapping EPR spectrum, Figure (a), recorded in methanol showed the multiline oxygen-centred radical signals centred near g ≈ 2.0, confirming the generation of reactive oxygen species. The spectrum does not look like a clean simple DMPO–OOH quartet which might be due to the possibility of DMPO–OH or mixed oxygen radical species. We have performed the radical scavenger test to further investigate the role of oxygen radical species. Isopropyl alcohol (IPA) and parabezoqionone (pBQ) as a $^{\cdot}$OH and $^{\cdot}$O2 scavengers respectively. as a $^{\cdot}$OH and $^{\cdot}O_2$ scavengers respectively. Figure (b) shows that when TMB oxidation was performed in the presence of IPA there is small decrease in the oxidase activity was observed. However, when the TMB oxidation was performed in the presence of pBQ, Figure (b), the complete inhibition of oxidation reaction was observed. Therefore, it can be inferred that O_2 radicals play the major role in the oxidase-mimic activity of CeO_{2-x} NSs.

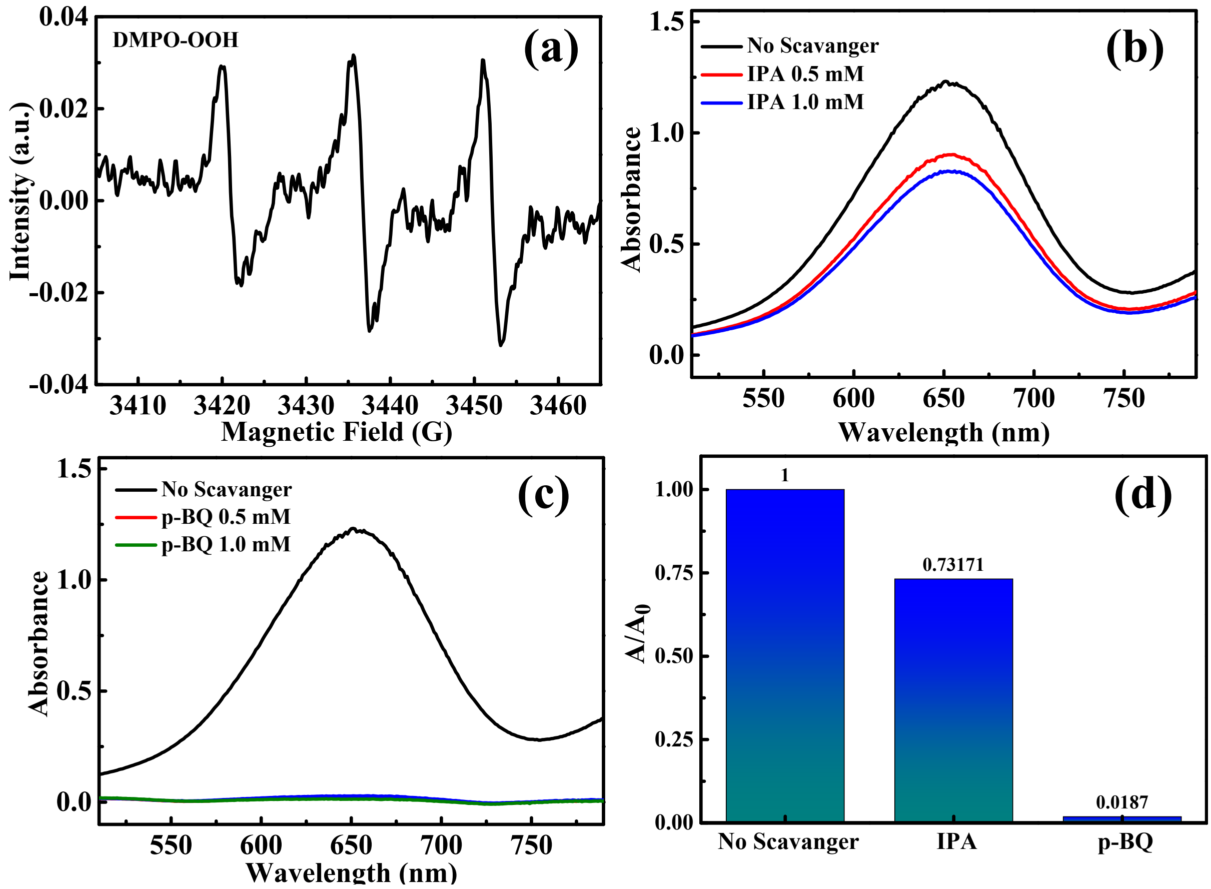

Figure S5: (a) EPR spectra of DMPO-MeOH, (b) and (c) show UV-vis absorption spectra of TMB oxidation monitored in the presence of IPA and pBQ respectively, (d) relative activity in the absence and presence of scavengers

The EPR spectra of CeO_2 NSs and of CeO_{2-x} NSs powder samples are shown in the following figure, with CeO_{2-x} NSs exhibiting a characteristic and relatively intense EPR signal. The EPR spectra exhibited a signal centred at 3443 G, g = 1.971, which may be due to the presence of Ce^{3+} paramagnetic centres formed after the reduction of Ce^{4+}.[22, 23]

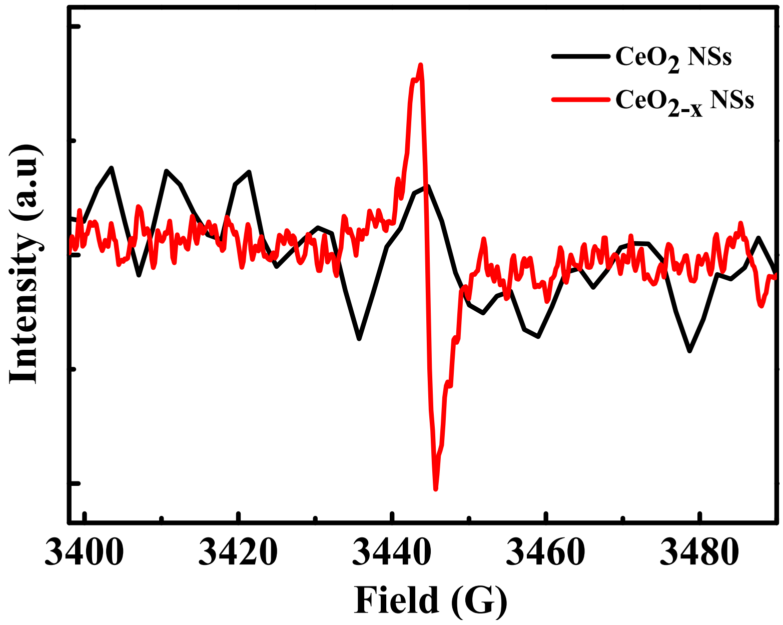

Figure S6: EPR spectra of CeO_2 NSs and of CeO_{2-x} NSs

Reusability and Stability analysis: To assess the reusability of the used sample, the catalyst was recovered after performing the various oxidation reactions. The recovered catalyst was washed several times with deionized water and ethanol through centrifugation and decantation and dried for 24 hours in a hot air oven at 50 °C. The recovered catalyst was then used to perform the next cycle of experiments. The catalyst stability was assessed by performing the XRD and XPS analysis of the recovered catalyst. We have repeated the sensing experiment for three consecutive cycles. The results show that after three cycles, the relative change in absorbance was decreased by about 6%. Furthermore, the XRD and XPS analysis of pristine and recovered catalysts shows no appreciable changes. Therefore, the CeO_{2-x} NSs show acceptable stability after 3 cycles. Also, we have added the results of the changes in catalytic activity during the storage life of the catalyst. The results show that the catalyst retains nearly 93% of its original activity after 5 months of storage, marking its usefulness for a longer time after the synthesis.

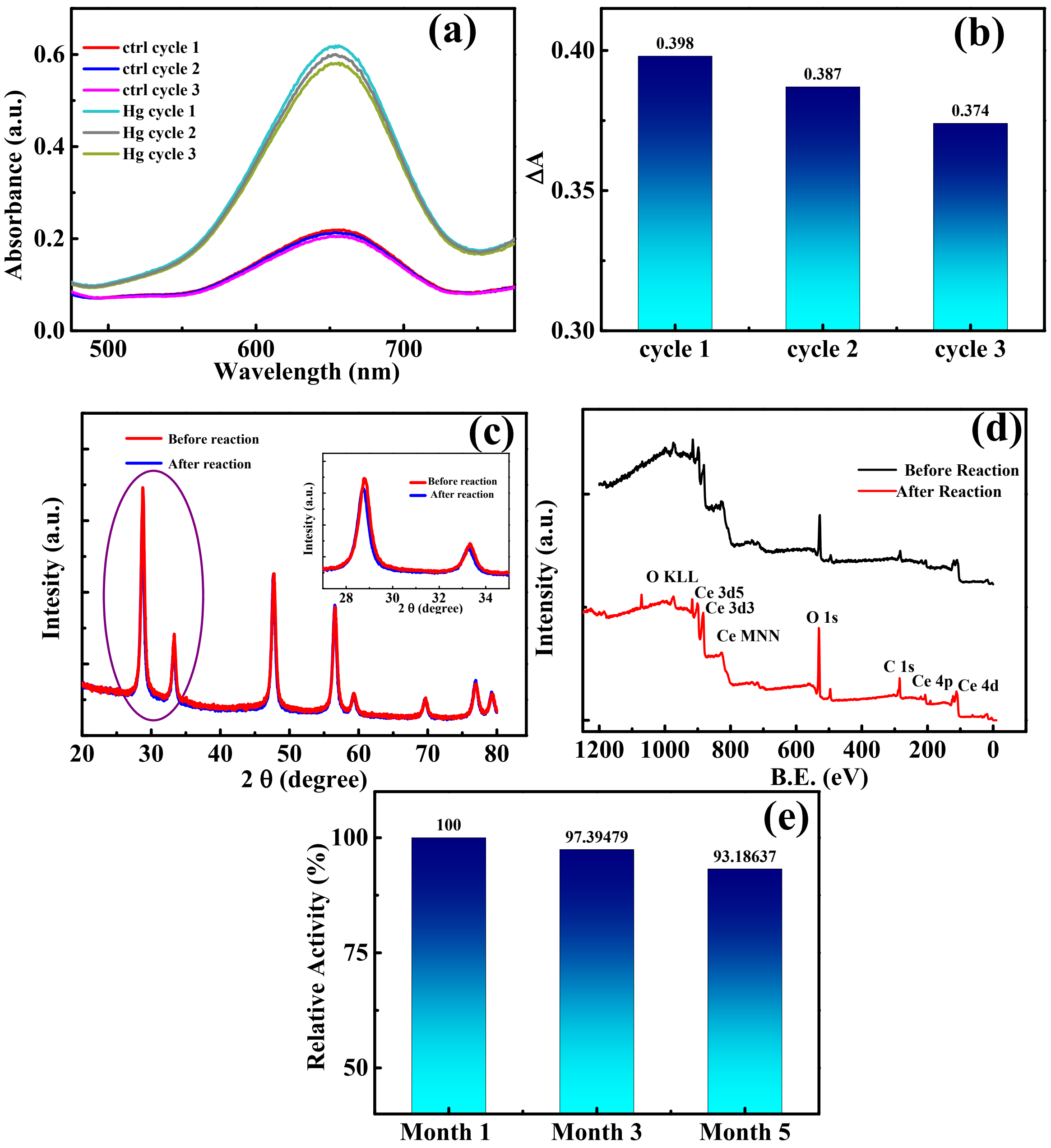

Figure S7: (a) UV-visible spectra ox TMB in the presence (labelled as Hg Cycle1-3) and absence (labelled as ctrl Cycle1-3) of Hg^{+2} for three consecutive cycles, and (b) corresponding changes in absorbance for each cycle, (c) the XRD data (d) XPS spectra of pristine and recovered CeO_{2-x} NSs, (e) Relative activity analysis on the Storage a life of catalyst

Reference:

1. Choudhury, B.; Chetri, P.; Choudhury, A., Annealing temperature and oxygen-vacancy-dependent variation of lattice strain, band gap and luminescence properties of CeO2 nanoparticles. *Journal of Experimental Nanoscience* **2015,** *10* (2), 103-114.
2. Kong, M.; Li, Y.; Chen, X.; Tian, T.; Fang, P.; Zheng, F.; Zhao, X., Tuning the relative concentration ratio of bulk defects to surface defects in TiO2 nanocrystals leads to high photocatalytic efficiency. *Journal of the American Chemical Society* **2011,** *133* (41), 16414-16417.
3. Sungu Akdogan, C. Z.; Gokcal, B.; Polat, M.; Hamaloglu, K. O.; Kip, C.; Tuncel, A., Porous, oxygen vacancy enhanced CeO2–x microspheres with efficient enzyme-mimetic and photothermal properties. *ACS Sustainable Chemistry & Engineering* **2022,** *10* (29), 9492-9505.
4. Asati, A.; Santra, S.; Kaittanis, C.; Nath, S.; Perez, J. M., Oxidase-like activity of polymer-coated cerium oxide nanoparticles. *Angewandte Chemie* **2009,** *121* (13), 2344-2348.
5. Cheng, H.; Lin, S.; Muhammad, F.; Lin, Y.-W.; Wei, H., Rationally modulate the oxidase-like activity of nanoceria for self-regulated bioassays. *Acs Sensors* **2016,** *1* (11), 1336-1343.
6. Kumar, A.; Singh, R.; Pathak, A.; Guin, D.; Tripathi, C. S. P., CeO2 nanosheets with prominent peroxidase-mimicking activity for the colorimetric sensing of H2O2, glucose, and ascorbic acid. *Next Nanotechnology* **2025,** *8*, 100239.
7. Kahandal, A.; Sharma, L.; Sirdeshmukh, V.; Kulkarni, A.; Tagad, C., A sensitive image-based optical detection of heavy metal ions using green synthesized silver nanoparticles. *International Journal of Environmental Science and Technology* **2023,** *20* (8), 9077-9088.
8. Chen, L.; Fu, X.; Lu, W.; Chen, L., Highly sensitive and selective colorimetric sensing of Hg2+ based on the morphology transition of silver nanoprisms. *ACS Applied Materials & Interfaces* **2013,** *5* (2), 284-290.
9. Zhu, J.; Zhao, B.-z.; Qi, Y.; Li, J.-J.; Li, X.; Zhao, J.-W., Colorimetric determination of Hg (II) by combining the etching and aggregation effect of cysteine-modified Au-Ag core-shell nanorods. *Sensors and Actuators B: Chemical* **2018,** *255*, 2927-2935.
10. Wang, S.; Zhang, H.; Wang, L.; Huang, C.; Ma, J.; Wang, L.; Wang, X.; Chen, L., Portable smartphone assisted multi-color colorimetric sensor for the detection of mercury ions based on the peroxidase-like activity of platinum nanoparticles. *Analyst* **2026**.
11. Zhao, X.; Li, S.; Yu, X.; Gang, R.; Wang, H., In situ growth of CeO 2 on gC 3 N 4 nanosheets toward a spherical gC 3 N 4/CeO 2 nanozyme with enhanced peroxidase-like catalysis: a selective colorimetric analysis strategy for mercury (II). *Nanoscale* **2020,** *12* (41), 21440-21446.
12. Li, J.; Li, L.; Bi, X.; Liu, X.; Luo, L.; You, T., Fluorescence/colorimetry dual-mode sensing strategy for mercury ion detection based on the quenching effect and nanozyme activity of porous cerium oxide nanorod. *Sensors and Actuators B: Chemical* **2022,** *360*, 131483.
13. Chen, Z.; Zhang, Z.; Qi, J.; You, J.; Ma, J.; Chen, L., Colorimetric detection of heavy metal ions with various chromogenic materials: Strategies and applications. *Journal of hazardous materials* **2023,** *441*, 129889.
14. Yang, M.; Wang, J.; Xue, X.; Jiang, H., Colorimetric detection of Hg 2+ based on the enhanced oxidase-mimic activity of CuO/Au@ Cu 3 (BTC) 2 triggered by Hg 2+. *RSC advances* **2024,** *14* (20), 13808-13816.
15. Wang, J.; Shen, M.; Meng, F.; Han, X.; Zhang, M., A portable paper-based analytical device mediated by transition metal selenide nanozymes based on Hg2+-activated oxidase-like activity. *Chemical Engineering Journal* **2025,** *512*, 162683.
16. Das, T.; Borah, P.; Saikia, M.; Dutta, P.; Das, M. R., Transforming coal byproducts into a novel oxidase-mimetic nanozyme for colorimetric detection of mercury ions. *ACS Sustainable Resource Management* **2025,** *2* (8), 1379-1389.
17. Liu, Z.; Niu, R.; Li, M.; Li, Z.; Guo, Y., A colorimetric and electrochemical dual-mode Hg2+ sensor utilizing oxidase-like activity arising from the combination of Hg2+ and palladium metal-organic framework@ graphene. *Microchimica Acta* **2024,** *191* (6), 352.

18. Lou, C.; Yang, F.; Zhu, L.; Sun, Q.; Yang, Y.; Guo, J., Dual-function sensor based on NH2-MIL-101 (Fe)@ Cu/CeO2 nanozyme for colorimetric and fluorescence detection of heavy metals. *Colloids and Surfaces A: Physicochemical and Engineering Aspects* **2023,** *677*, 132398.
19. Harshavardhan, A.; Nagaveni, V.; Madhu, H., Optical, dye degradation, and electrochemical sensor studies of CeO2 and CuO nanoparticles by pomegranate peel. *Tetrahedron Green Chem* **2025,** *5*, 100077.
20. Magdalane, C. M.; Kaviyarasu, K.; Vijaya, J. J.; Siddhardha, B.; Jeyaraj, B.; Kennedy, J.; Maaza, M., Evaluation on the heterostructured CeO2/Y2O3 binary metal oxide nanocomposites for UV/Vis light induced photocatalytic degradation of Rhodamine-B dye for textile engineering application. *Journal of Alloys and Compounds* **2017,** *727*, 1324-1337.
21. Al-Wafi, R.; Hammad, M. S.; Mansour, S., Superior dye degradation for wastewater treatment based on nanocomposites of cerium oxide, gadolinium oxide and graphene oxide. *Ceramics International* **2024,** *50* (10), 16736-16746.
22. Araújo, V.; Avansi, W.; de Carvalho, a. H.; Moreira, M.; Longo, E.; Ribeiro, C.; Bernardi, M. I. B., CeO 2 nanoparticles synthesized by a microwave-assisted hydrothermal method: evolution from nanospheres to nanorods. *CrystEngComm* **2012,** *14* (3), 1150-1154.
23. Ratnasamy, P.; Srinivas, D.; Satyanarayana, C.; Manikandan, P.; Kumaran, R. S.; Sachin, M.; Shetti, V. N., Influence of the support on the preferential oxidation of CO in hydrogen-rich steam reformates over the CuO–CeO2–ZrO2 system. *Journal of Catalysis* **2004,** *221* (2), 455-465.
